# Contribution of Nucleus Accumbens Core (AcbC) to Behavior Control during a Learned Resting Period: Introduction of a Novel Task and Lesion Experiments

**DOI:** 10.1371/journal.pone.0095941

**Published:** 2014-04-28

**Authors:** Chika Sato, Masato Hoshino, Naori Ikumi, Kentarou Oba, Akiko Koike, Osamu Shouno, Tatsuhiko Sekiguchi, Tetsuya Kobayashi, Takeo Machida, Gen Matsumoto, Hiroyuki Furudate, Tetsuya Kimura

**Affiliations:** 1 Laboratory for Brain-Operative Expression, RIKEN Brain Science Institute, Wako, Saitama, Japan; 2 Area of Regulatory Biology, Division of Life Science, Graduate School of Science and Engineering, Saitama University, Saitama, Saitama, Japan; 3 Honda Research Institute Japan Co., Ltd., Wako, Saitama, Japan; 4 Laboratory for Alzheimer's Disease, RIKEN Brain Science Institute, Wako, Saitama, Japan; 5 Department of Aging Neurobiology, National Center for Geriatrics and Gerontology, Obu, Aichi, Japan; Centre national de la recherche scientifique, France

## Abstract

In recent years, the study of resting state neural activity has received much attention. To better understand the roles of different brain regions in the regulation of behavioral activity in an arousing or a resting period, we developed a novel behavioral paradigm (8-arm food-foraging task; 8-arm FFT) using the radial 8-arm maze and examined how AcbC lesions affect behavioral execution and learning. Repetitive training on the 8-arm FFT facilitated motivation of normal rats to run quickly to the arm tips and to the center platform before the last-reward collection. Importantly, just after this point and before confirmation of no reward at the next arm traverse, locomotor activity decreased. This indicates that well-trained rats can predict the absence of the reward at the end of food seeking and then start another behavior, namely planned resting. Lesions of the AcbC *after* training selectively impaired this reduction of locomotor activity after the last-reward collection without changing activity levels before the last-reward collection. Analysis of arm-selection patterns in the lesioned animals suggests little influence of the lesion in the ability to predict the reward absence. AcbC lesions did not change exploratory locomotor activity in an open-field test in which there were no rewards. This suggests that the AcbC controls the activity level of planned resting behavior shaped by the 8-arm FFT. Rats receiving training after AcbC lesioning showed a reduction in motivation for reward seeking. Thus, the AcbC also plays important roles not only in controlling the activity level after the last-reward collection but also in motivational learning for setting the activity level of reward-seeking behavior.

## Introduction

The observation that neural activity can increase during awake and intentional resting states has received increasing research attention in recent years, from examination of individual neurons to functional brain imaging to behavior. Examples of intentional resting states at the behavioral level include periods when a subject is not performing a task, times when a subject is in task-residuals [1], when no training stimuli are present [2], or when a subject is completely at rest [3]. Functional imaging studies have demonstrated that many brain regions show enhanced activity during resting states compared to activity during task performance [2], [4]–[6]. In addition, patterns of correlated brain activity during rest are consistent among individuals [7], [8].

A network of brain regions that exhibit increased activity during rest and decreased activity during a given task has been identified. This network has been termed the “default mode network” (DMN) [4]. Enhanced DMN activity is detectable when subjects lie quietly with their eyes closed, during low-demand tasks, and during the inter-trial interval in high-demand tasks [2], [5], [6], [9]. Importantly, the patterns of correlated brain activity during rest can predict the disease progression of patients with mild cognitive impairment [10], [11]. From these observations, it is hypothesized that enhanced neural activity during rest involves non-random cognitive processing. It is still unclear, however, what the physiological function is of this brain activity during rest.

In rats, neural activity is often elevated during a no-task condition or resting state. Taha and Fields, for example, showed that neurons in the nucleus accumbens (Acb) display enhanced activity when the rat fails to exhibit a learned reward-approach behavior in a no-task state. [12]. The Acb serves as an important interface between the limbic and motor systems [13]–[15], since it receives inputs from the medial prefrontal cortex/cingulate cortex, the substantia nigra, and hippocampus and provides outputs to the substantia nigra and medial dorsal nucleus of thalamus. A fMRI study [3] showed that Acb activity during resting states reflects functional connectivity with the ventral anterior cingulate cortex, an important pivotal region in the DMN of the human brain [4], [16]. On the basis of these findings, the Acb is expected to mediate some important functions of brain activity in a resting state, which is within the scope of the DMN.

Acb function mainly has been investigated by selective lesion techniques [17]–[21] targeting the entire Acb or its subdivisions: Acb shell (AcbS) and Acb core (AcbC) [22]. Lesions of the AcbC prevent the inhibition of impulsive behavior [17], suggesting that the AcbC contributes to instrumental learning when long delays intervene between subjects' actions and ensuing outcomes that reinforce behavior. Disruption of dopaminergic (DA) function in the Acb also alters effort-related decision-making in choice procedures that involve allocating responses among different reinforcement values and response costs [23]–[27]. Thus, the Acb, especially the AcbC, plays a special role in behavior control in relation to behavioral cost and/or expected reward value. Also, electrical stimulation of the Acb has been reported to inhibit sucrose consumption [28]. Thus, Acb activity observed mainly in resting states could effectively be a gating mechanism for objective-oriented behavior, expressed by inhibiting or disinhibiting behavioral control. It is, however, still unclear how neural activity during these resting periods influences the optimization process of objective-oriented behavior or decision-making based on behavioral cost.

In the present study, we introduced a behavioral task called the 8-arm food foraging task (8-arm FFT), a learning and memory task in which the reward condition changes (from reward-available to reward-unavailable situations). In the 8-arm FFT, animals learn how to predict, without external cues, the reward condition and learn when to voluntarily change their activity level according to changes in the reward condition. The active (reward-available) and resting (reward-unavailable) periods were specifically built into the task to better understand the roles of different brain regions in the regulation of behavioral activities in an arousing or in a resting period. We made selective AcbC lesions in three different conditions. First, we examined how the AcbC contributes to the control of motor activity during active and resting periods by lesioning animals after training on the 8-arm FFT. Second we examined how the AcbC contributes to shaping optimized motor behavior through repetitive training by lesioning the AcbC before training on the 8-arm FFT. Finally, to understand the nature of the behavior inhibited by AcbC activity, we examined the effect of AcbC lesions on general motor activity or simple exploration.

## Materials & Methods

### Animals

This study was carried out in strict accordance with the Guidelines for the Care and Use of Experimental Animals at Saitama University, Japan. All protocols for animal experiments were approved by the Saitama University Institutional Animal Care and Use Committee (permit number: H21-1-16). Subjects were adult male Long-Evans rats (Tokyo Laboratory Animals Science CO., Ltd, Japan) weighing 300–360 g. They were 5–6 months old at the beginning of behavioral testing. All rats were given ad libitum access to water and were handled daily for 10–15 min. Food intake of each rat was restricted: Rats were given daily 12–16 g of solid food pellets (CRF-1, CLEA Japan, Inc.) in order to maintain their weight at 85% of their free-feeding weight. This was done in order to motivate the animals to obtain the food reward in the behavioral task.

#### Experimental groups

The various experimental groups are explained in detail in the Methods S1.

### AcbC lesions

Stereotactic lesions of the AcbC were made using a standard stereotaxic apparatus (David Kopf Instruments). Rats were first injected with pentobarbital sodium (i.p.; 50 mg/kg body weight, Dainippon Sumitomo Pharma Co., Ltd) dissolved in saline and then maintained on anesthesia with isoflurane (Foren; Abbott Japan Co., Ltd.) for the surgical procedure. Bilateral lesions were made by infusing ibotenic acid (0.6 µl/hemisphere of 5 mg/ml ibotenic acid in PBS; Sigma Aldrich, Japan) through a stainless steel needle (200 µm outer diameter) attached to the stereotaxic device. Coordinates for injection sites were as follows: AP, +1.9 mm rostral to bregma; ML, ±1.7 mm lateral to the midline; V, −6.3 mm ventral to the dura mater. Sham-lesioned rats received injections of the same volume (0.6 µl/hemisphere) of PBS at these coordinates. The needle was left in place for 15–20 min to allow the toxin to diffuse sufficiently within the target sites. Rats were allowed to recover for 7 days following the lesion surgery. For the first 4 days after surgery, rats were given daily i.p. injections of antibiotic solution (0.4 ml/day of 1 mg/ml gentamicin in saline; Schering-Plough).

### Behavior analysis and task control

#### Maze

An eight-arm radial maze was used for the 8-arm FFT. The maze had an octagonal-shaped center platform (40 cm across), from which eight equally spaced arms radiated outward; each arm measured 50 cm×9 cm. Throughout this report, we refer to the end of the arm farthest away from the maze center as the “tip” and the center platform as the “platform.” A food cup was located at the tip of each arm.

Eight computer-controlled gates (clear 9×13 cm acryl plates covered with clear blue plastic skirts) situated between the platform and the beginning of the arms controlled entry to the arms. The maze was elevated 40 cm above the floor and was centered in a room (4×5×3 m^3^) with black walls and numerous external landmarks (e.g., flat pictures and three-dimensional objects). The room ceiling was 180 cm from the maze surface. In preliminary studies, we observed that rats trained in a room with a higher ceiling (250 cm from the maze surface) occasionally showed a random pattern of arm selection after 25 trials (data not shown; regarding the definition of arm-selection pattern, see *Analysis of arm-visit patterns* under *Data analysis*). We believe that the higher ceiling induced more anxiety in the rats, causing more re-entry arm errors during the reward-available period. Thus, all of our data in the present study were collected in the lower-ceiling room (i.e., 180 cm from the maze surface). Four recessed lights (7 lux at the platform surface) provided indirect illumination, and noise was presented to mask extraneous noise. These environmental conditions ensured that rats reliably performed sequential pattern of arm selection.

#### Open field

The open-field apparatus consisted of a circular arena made of transparent acrylic plastic. Its dimensions were 60 cm in diameter by 50 cm in height. To provide a different environment and reward context from the one used in the 8-arm FFT, the open-field arena was elevated 70 cm above the floor in the testing room. The former environment was associated with a place where the animals could acquire a reward and the latter had no such association. Eight recessed lights (about 20 lux at the center of the arena) provided indirect illumination. Noise was presented to mask extraneous noise.

#### Movement tracking system

Rats' movements on the maze were recorded by a CCD camera mounted 150 cm above the maze surface. Video data (440 pixels×440 pixels) were digitized (Meteor II A/D board; Matrox Electronic Systems, Ltd.) at a rate of 30 frames/sec and were analyzed in real time with custom software running on a PC. The rat's position (center of the rat's black head) in each frame was determined using graphic analysis functions (Matrox Imaging Library; Matrox Electronic Systems, Ltd.); the calculated position for each frame was stored onto a hard disk of the PC, along with the timing of gate-openings and the timing of each reward collection at the food cups. Reward sensors at the arm tips monitored the state of each food cup (filled vs. empty). The computerized tracking system also directed the task schedule for each trial by controlling the arm entry gates (opening/closing). These event signals were also simultaneously recorded onto the disk along with the time-stamps associated with the tracking data.

#### Behavioral tasks

8-arm FFT training: Rats received one trial per day. Prior to training in the 8-arm FFT, however, all animals received a preliminary learning trial, during which sucrose pellets (1811555(5TUT); PMI Certified Rodents TestDiet, Richmond, IN, USA) were liberally placed throughout the surface of each arm, from the beginning of the arms to the tips, and on the platform near the beginning of each arm. In this preliminary learning trial, the rat was first placed on the platform for 2–3 min while all arm gates remained closed. Then, all of the gates opened simultaneously, allowing access to the arms. About 30 min after the start of this preliminary learning trial, we stopped recording the behavior and returned the rat to its home cage.

**Figure 1:**
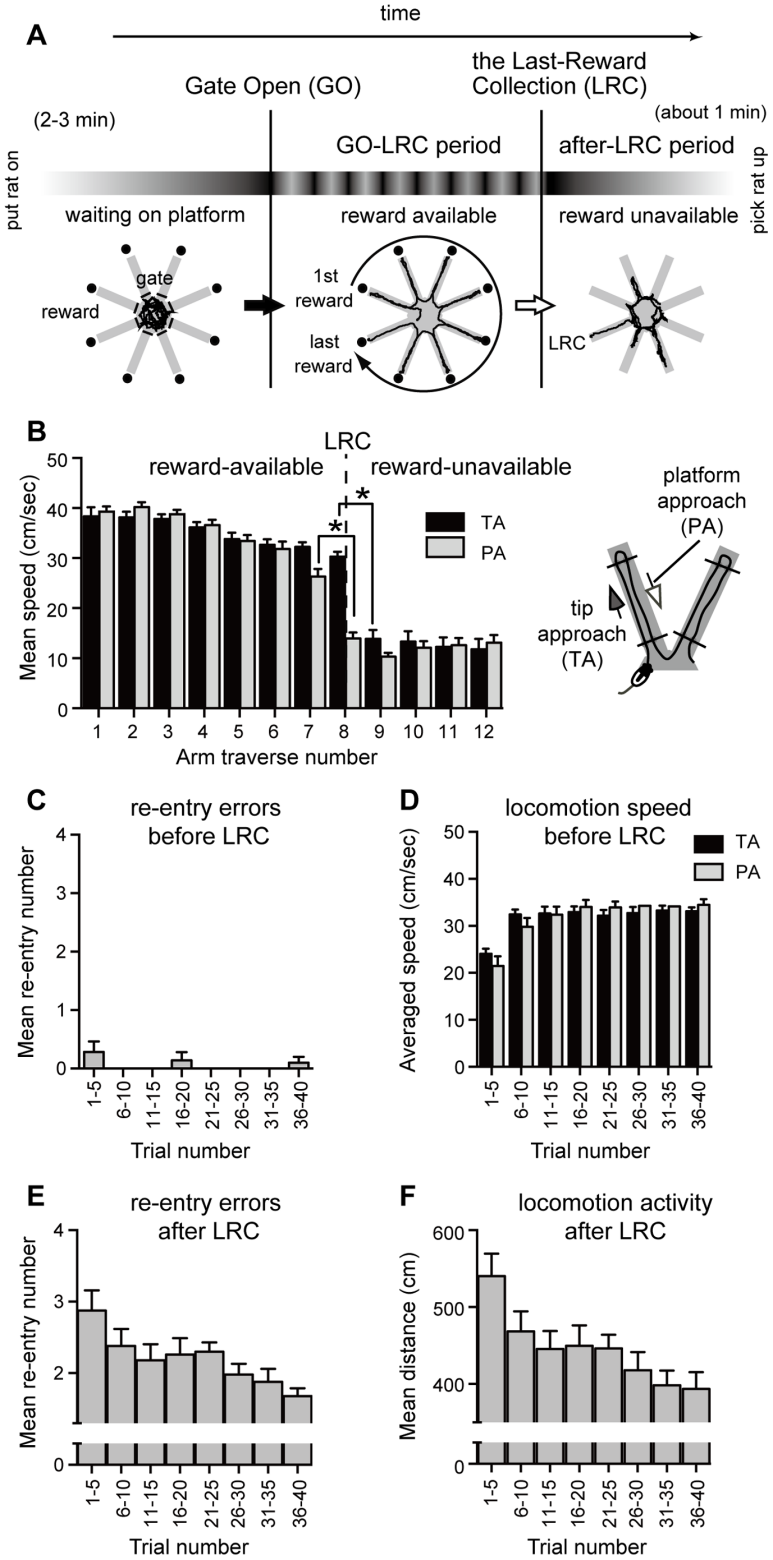
Introduction of the 8-arm food foraging task and performance of normal adult rats. **A**: Schematic design of the 8-arm food foraging task (8-arm FFT). Horizontal bar shows the sequence of task phases and critical events during a trial. Each rat was placed on the platform with all arm gates closed (before-GO period). Two to three minutes later, all gates were opened (GO), allowing free access to the tip of each arm, where the reward was located in a food cup. Five minutes after the last-reward collection (LRC), the rat was removed and placed in its home cage. Bottom row of diagrams in (A) shows movement traces during the different phases. Each of the solid, irregular lines shows a typical example of well-trained rat movements (41st–45th trials). Repeated training produced a pattern of reliable and low-cost behavior, which was characterized by automatic-appearing sequential arm selection before LRC. After the LRC (reward-unavailable period), random patterns were common, with much slower running speeds compared to before the LRC. **B**: Timing of changes in mean tip-approaching (TA, black filled bars) and platform-approaching speed (PA, open bars) of well-trained rats (41st–45th trials). The platform-approaching speed at the 8th approach (just after LRC) was significantly slower than that of the 7th approach (one-way ANOVA, *F*_11,156_ = 100.8, *P*<0.05; Dunn-Sidak test, * *P*<0.05). Similar to platform approaching, a significant slowing of tip-approaching speeds occurred between the 8th and 9th approaches (*P*<0.05). Dashed line shows at which point the LRC occurs. **C**: Change in the number of arm re-entry errors before LRC (reward-available period). **D**: Change in average tip-approaching and platform-approaching speeds before LRC. **E**: Change in the number of arm re-entry errors for one minute after LRC. **F**: Change in traveling distance for one minute after LRC. The various experimental groups are explained in detail in the Supplementary Methods. Error bars denote SEM.

For the 8-arm FFT training, two sucrose pellets were placed in each food cup at the arm tips (Figure 1). As with the preliminary learning trial, the rat was placed on the platform of the maze for 2–3 min with the gates closed. The gates then opened simultaneously (gate-open, GO; Figure 1A), allowing the rat to collect the eight rewards from the arm tips. An error was defined as re-entering an arm from which the rat already collected the reward. After the rat acquired the final or last reward (last-reward collection, LRC), we continued recording behavioral data for at least 1 min. During this reward-unavailable period after the last-reward collection, all the gates remained open, and the rat was allowed to freely traverse up and down the maze arms and the platform, if it desired. It was then returned to its home cage.

For some trials, or portions of trials, we occasionally failed to obtain behavioral data because of an instrument malfunction. These rare trials were counted as having “no data,” and were excluded from analysis.

Open-field test: The rat was placed into the center of the arena and allowed to move freely around the open-field and explore the environment. The rat's traveling distance was recorded during a test period of 30 min using the same tracking system that was used in the radial 8-arm FFT. The arena floor was cleaned before the next rat began its open-field test.

#### Data analysis

Analysis of motor activity: For behavioral analyses, we calculated the rats' traveling distance before and after the last-reward collection, the tip-approaching speeds, and the platform-approaching speeds on each arm approach for each trial. We also measured the number of arm re-entry errors for each trial. Traveling distance in a trial was calculated from tracking data, which recorded the rat's maze position every 32 msec. The timing of the last-reward collection was estimated from data obtained from reward sensors. Tip-approaching speed was defined as the rat's mean speed from arm entry to reaching the end of the arm (distance covered divided by time to reach the tip). The calculation started when the rat's head passed over a virtual line at the beginning of the arm (5 cm from the center of the platform) to when it reached the end of the arm and stopped. Platform-approaching speed was defined as the rat's mean speed as it moved away from the end of the arm to the maze center. This calculation started when the rat's head passed over a virtual line at the end (5 cm from the end of the arm, or if the rat did not reach the end of the arm, the point at which the rat turned around) to the point at which the rat's head passed over a virtual line at the platform (5 cm from the border between the arm and the center of the platform). The mean speeds for each rat in five successive trials were analyzed for each arm. These mean speeds were averaged before the last-reward collection in order to compare performance of rats with AcbC lesions and rats with sham lesions during the reward-available period. Tallying the number of re-entry errors in each trial was also done automatically from the same calculation done for the tip-approaching speed.

**Figure 2:**
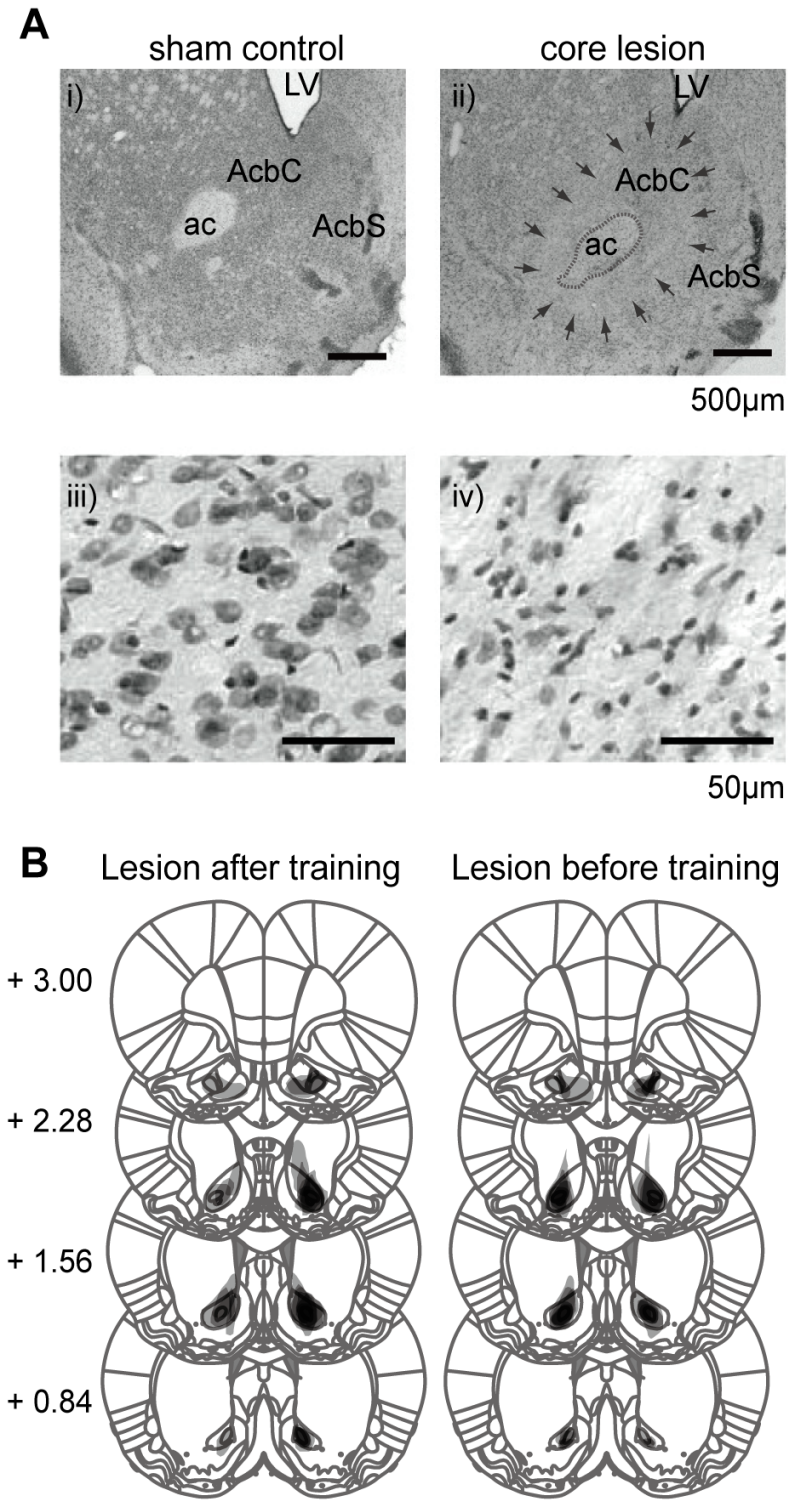
Histological analysis of AcbC-lesioned rats. Extent of ibotenic acid AcbC lesions in rats for two sets of experiments: before the start of repetitive training on the 8-arm FFT (n = 6) and after repetitive training (n = 6) **A**: Representative photomicrographs of Nissl-stained coronal sections showing the AcbC in sham control and lesioned rats. Bidirectional reduction of neuronal components is present around the ibotenic acid injection site (ii). Sham control rats showed very little destruction around the AcbC (i). Higher magnification images in iii and iv. AcbC, nucleus accumbens core; AcbS, nucleus accumbens shell; ac, anterior commissure; LV, lateral ventricle. **B**: Composite drawings of the extent of lesioned areas (filled gray) in standardized coronal drawings as determined in all behaviorally assessed rats. Darker gray areas indicate overlap of lesions among rats. Numbers represent the rostrocaudal distance (mm) from bregma. Left column of sections shows the size and location of the lesioned areas in rats receiving an AcbC lesion *after* training (n = 6), whereas the right column shows the size and location of the lesioned areas in rats receiving an AcbC lesion *before* training (n = 6). Plates were adapted from the atlas of Paxinos and Watson [29].

Analysis of prediction of reward absence: We were interested in determining whether rats showed behavioral signs of knowing when the trial was complete, even before they confirmed there would be no more rewards (i.e., predicted no reward would be present after 8 correct arm choices). Selective differences in certain task behavioral parameters on the 8-arm FFT, before and after AcbC lesions, might provide evidence of its role in organizing/regulating motor activity control. Several behavioral parameters were analyzed for this purpose: (1) platform-approaching speed just before (7th platform approach) and after (8th platform approach) the last-reward collection (see Figure 1B), (2) tip-approaching speed just before (8th tip approach) and after (9th tip approach) the last-reward collection, and (3) occurrence of a random pattern of arm selection on an attempted 9th arm visit. We observed that well-trained rats that had received an AcbC lesion exhibited increased speeds on the 8th platform approach similar to the speed on the 7th platform approach. For this reason, we used only the latter two parameters to estimate whether AcbC-lesioned rats knew that just after the 8th reward collection this was to be the last reward.

Analysis of arm-selection patterns: We were interested in determining whether rats displayed a sequential pattern of arm selection, or one that tended to be somewhat random in the 8-arm FFT. Of course for the first arm selection, the pattern of arm selection could not be determined. For the second arm selection, we regarded it as sequential if the rat traversed an arm adjacent to the first one selected, followed by another adjacent arm selection, continuing to the end, selecting each successive adjacent arm (Figure S1A). This means each successive turn performed by the rat was 45°.

We regarded the pattern of arm selection as a non-sequential (“random”) one if at some point in the trial the rat selected and ran down an arm non-adjacent to the prior one selected. In the example shown (Figure S1B), the 3rd arm visited by the rat is two arms away from the 2nd arm visited (i.e., 90° turn), and the 8th arm visited is four arms away from the 7th arm visited (i.e., 180° turn). A rat showing this pattern, or one similar, would have a non-sequential pattern of arm selection.

Analysis of random pattern of arm selection: To determine a rat's first random arm-selection pattern, we counted the number of first random pattern of arm selections at each arm traverse of the rat in five successive trials.

Statistical comparisons: We used one-way ANOVA to examine group differences in traveling speeds (arm tip-approaching and platform-approaching speeds) along the arms in a trial of the 8-arm FFT. Dunn-Sidak post hoc tests were used to determine where any overall speed differences detected in the ANOVA occurred in the sequence of arm visits in the trial. When appropriate, comparisons between rats with AcbC lesions and sham control rats were tested with unpaired t-tests. To test for an occurrence of a random pattern of arm selection on a potential 9th arm selection, we compared the data in the same well-trained rats before and after they received AcbC lesions by using the Chi-square test. To examine the relationship between platform-approaching speed during the reward-available period and locomotor activity (distance traveled) after the last-reward collection, we used correlation analysis. Statistical analyses were performed using GraphPad Prism 6.0 for Mac (GraphPad Software, La Jolla, CA) and MATLAB (Mathworks, Natic, MA).

#### Histology

After the end of the behavioral experiments, rats were deeply anesthetized with pentobarbital sodium and perfused transcardially with 0.01 M PBS followed by 10% formaldehyde. The brains were removed and postfixed in 10% formaldehyde and then cryoprotected by placing them in a solution of 30% sucrose in 0.01 M PBS for 48–72 h at 4°C until they equilibrated (i.e., sank to the bottom). Coronal sections (40 µm thick) were cut on a freezing microtome and collected in cold 0.1 M PBS. Sections were mounted onto silane-coated glass slides and stained with cresyl violet (Raymond A. Lamb, Ltd.). Stained sections containing the anterior end to the posterior end of the AcbC were examined using a light microscope by an investigator who was blind to the behavioral results. Areas of neuronal loss were mapped onto drawings of standardized coronal sections of the rat brain [29]. The control rats had no detectable histological damage from the PBS injections.

We used ImageJ software (developed at the U.S. National Institutes of Health and available on the Internet at http://rsb.info.nih.gov/ij/) to estimate and compare the extent of the lesioned areas across all rats. Rats with unilateral AcbC lesions did not differ behaviorally from rats with bilateral AcbC lesions. Next, we determined the percentage of the AcbC that was lesioned by using ImageJ to measure the mapped areas of the lesions in the individual drawings of standardized coronal sections at different posteroanterior levels. We chose the area measurement of the largest lesion (either in left or right hemisphere) for the percentage calculations.

## Results

### Logical structure of the 8-arm FFT

The horizontal bar of Figure 1A schematically shows the sequence of task events and phases on the 8-arm FFT. The task could logically be divided into 3 movement phases: (1) waiting on platform before the gates open, (2) traverses on the maze arms and platform up to the last-reward collection (reward-available period), and (3) traverses *after* the last-reward collection (reward-unavailable period). During this last phase, the rat could freely traverse the platform and arms, if it desired. There were only 8 rewards to be retrieved during a trial, each in a food cup at the arm tip.

The task also has several critical events. The first important event for the rat—“gate-open” (GO)—is the simultaneous opening of all the gates after the waiting period (Figure 1A, first vertical line), indicating that rewards are available.

Another critical event occurs toward the end of the task, denoted by us as the “last-reward collection” (LRC; Figure 1A, second vertical line), the point when all 8 rewards have been retrieved. In the context of the gates being opened, this event differentiates a time when rewards are available from one when rewards are unavailable. For each trial after the LRC, the rat remained on the maze for at least 1 minute. This extended time after the last reward was collected (after-LRC period; Figure 1A) was expected to produce a reduction in a normal rat's locomotor activity, because there would be no more rewards until the next day's training. This, in turn, would contribute to the reduction of behavioral cost.

### Behavioral shaping in the 8-arm FFT of normal rats

Repetitive training on the 8-arm FFT resulted in progressive shaping of a stereotyped locomotor behavior (Figure 1A). With increased training trials, unlesioned adult rats (n = 10) optimized their movements, resulting in low-cost behavior. This was manifested by purposeful, rapid traverses up and down the arms and by a sequential pattern of arm selections (Figure 1A; GO-LRC period) up to the last-reward collection (LRC). After eating the last reward (Figure 1A; after-LRC period), the rats' pattern of arm selections tended to be random. It is important to note that rats received one trial per day (see Materials and Methods section).

After repetitive training, the rats' tip-approaching speeds and platform-approaching speeds of each arm were relatively high before the last reward was collected (dashed vertical line, Figure 1B), while after the last reward was collected, speeds in both directions were significantly slower (both *P*'*s*<0.05; Figure 1B). The platform-approaching speeds between the 7th and 8th approach were significantly different in well-trained rats (P<0.05). The decrease in tip-approaching speed occurred after the 8th approach (P<0.05; comparison between 8th and 9th approach; Figure 1B).

Importantly, the decrease in platform-approaching speed occurred just after the last-reward collection and before the rats' 9th arm selection. That is, this decrease in speed occurred before the rats actually confirmed that there were no more rewards. Thus, the slowing down in activity after the last critical event of the 8-arm FFT suggests that well-trained rats predicted that no more rewards were available and started the slow locomoting behavior, namely planned resting.

Details of the behavioral shaping process, or optimization of actions, in the 8-arm FFT are shown in Figures 1C-1F. The very rapid reduction of arm re-entry errors before the last reward was eaten (Figure 1C) shows that normal rats tended to employ a sequential pattern of arm selection within the first 5 trials (see subsection about sequential pattern of arm selection, Analysis of arm-selection patterns in Materials and Methods). Rats acquired all rewards without errors as long as they employed the sequential pattern of arm selection (Figure S2).

Once rats consistently used the sequential pattern of arm selection, other parameters gradually changed (i.e., mean tip-approaching and mean platform-approaching speeds before the last-reward collection, number of re-entry errors, and activity level after the last-reward collection; Figures 1D–1F). A performance plateau was reached within 30 trials. Thus, we defined a well-trained rat as one that (1) had received 30 trials or more, (2) displayed rapid running on the maze up to the last-reward collection, and (3) showed slower locomotion on the maze after eating the last reward, the last critical event of the 8-arm FFT.

### Well-trained rats with AcbC lesions fail to show the normal reduction in activity level after the last-reward collection

We examined the role of the AcbC in execution of already optimized behavior shaped by the 8-arm FFT (see subsection, *Lesion experiments in well-trained rats* in Methods S1). A large part of the AcbC was damaged by the ibotenic acid injections (Figure 2). Before AcbC or sham lesions, both groups of rats performed similarly. None of the behavioral parameters were significantly different between the two groups (all P's>0.05; Figure S3).

**Figure 3:**
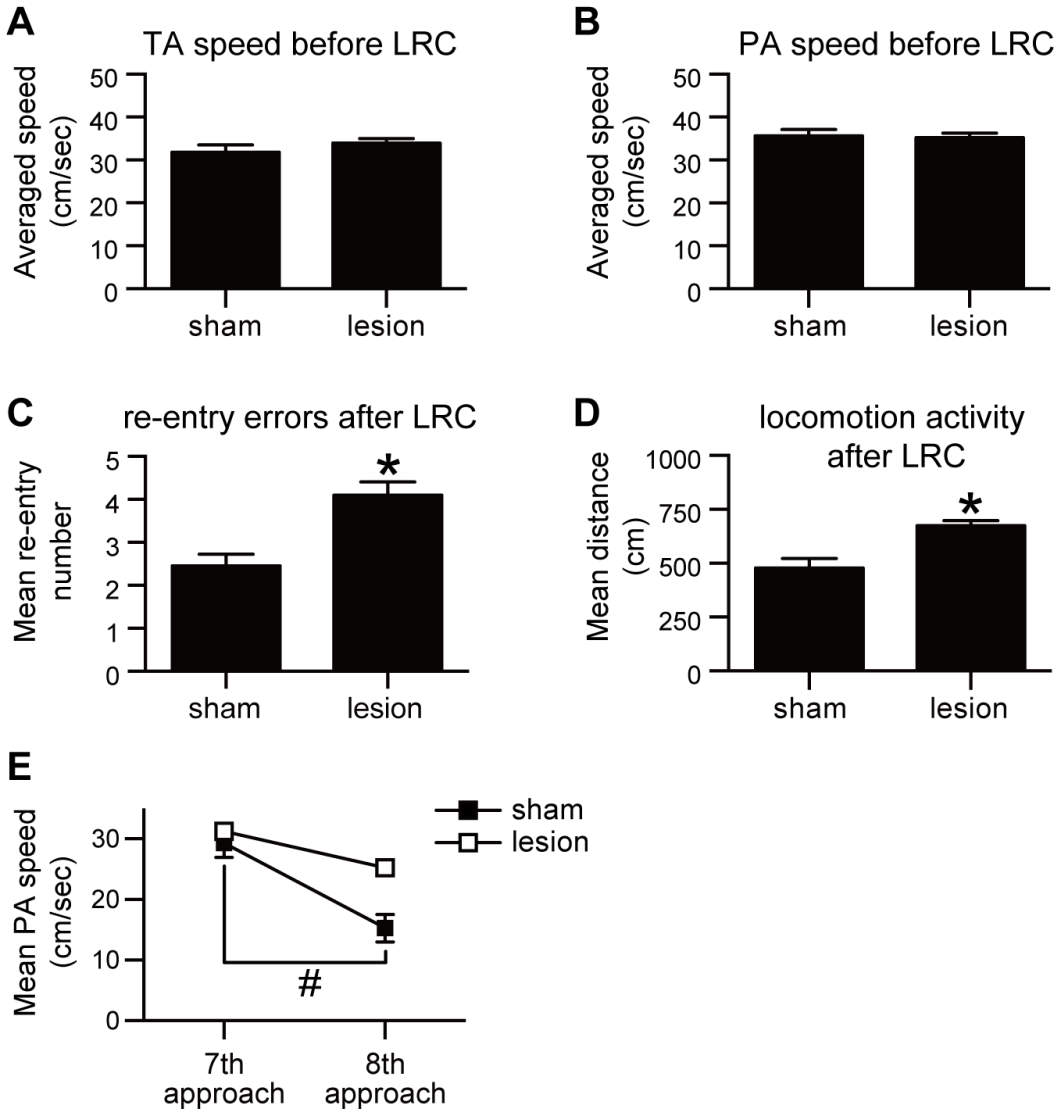
Effect of AcbC lesions on the execution of optimized behavior in the 8-arm FFT. AcbC (n = 6 rats) or sham control lesions (n = 6) were made in well-trained rats. **A**: Averaged tip-approaching (TA) speeds before the last-reward collection (LRC) did not differ in well-trained AcbC-lesioned rats compared to sham controls (t = 0.67, *P*>0.05, unpaired *t*-test). **B**: Averaged platform-approaching (PA) speeds before LRC in AcbC-lesioned rats also did not differ (t = 0.85, *P*>0.05, unpaired *t*-test). **C**: For the one min after the last reward was eaten (LRC), AcbC-lesioned rats had more re-entry errors compared to sham controls (t = 3.76, *P*<0.05, unpaired *t*-test). **D**: Rats with AcbC lesions showed increased locomotion activity after LRC (t = 4.47, *P*<0.05, unpaired *t*-test). **E**: Mean PA speed just before (7th arm visit) and just after (8th arm visit) LRC. In contrast to sham-lesioned rats, AcbC-lesioned rats did not significantly decrease their PA speed just after LRC (*F*_1,5_ = 28.27, *P*<0.05, two-way ANOVA; AcbC-lesions, *P*>0.05; sham-lesions, *P*<0.05, Sidak's multiple comparison test). *P<0.05 compared to sham controls; #*P*<0.05 compared to 7th arm visit. Error bars denote SEM. LRC, the last-reward collection; GO, gate-open; TA, tip-approaching; PA, platform-approaching.

By contrast, lesion of the AcbC in these well-trained animals produced an increase in the number of arm re-entry errors (Figure 3C), and an increase in the rat's activity after eating the last reward (Figure 3D) (P's<0.05). On the other hand, AcbC-lesioned rats showed no significant differences from the control rats in tip- and platform-approaching speeds before the last reward was retrieved (Figure 3A and B; P's>0.05). Moreover, AcbC-lesioned rats showed a sequential pattern of arm selection and few re-entry errors before the last reward collection, similar to sham controls (Figure S4A).

This lack of a normal decrease in speed in AcbC-lesioned rats after eating the last reward was confirmed in the 8th platform approach (Figure 3E). While control rats had significantly slower platform-approaching speeds between the 7th and 8th approach (P<0.05), speeds of AcbC-lesioned rats were statistically indistinguishable for these approaches (P>0.05). Thus, the AcbC appears to be involved in the prediction of reward unavailability, which then engages the planning of resting behavior, or the regulation of activity level during the resting period.

### AcbC lesions left intact the ability to predict the reward absence just after the last-reward collection

To examine the contribution of the AcbC on a rat's ability to detect when all rewards are retrieved, we analyzed three parameters that change between the period after the last-reward collection to before the rats reach an empty food cup (i.e., already rewarded) at the arm tip. These parameters are (1) platform-approaching speed just before (7th platform approach) and after (8th platform approach) the last-reward collection, (2) tip-approaching speed just before (8th tip approach) and after (9th tip approach) the last-reward collection, and (3) occurrence of a random pattern of arm selection on an attempted 9th arm visit. Although well-trained rats selected each arm sequentially before the last reward was retrieved, a random selection (see subsection about definition of random pattern of arm-selection, *Analysis of arm-selection patterns* in Materials and Methods) often occurred just after the last-reward collection (that is, at their 9th arm selection).

AcbC lesions in well-trained rats did not produce in subsequent trials an increase in random pattern of arm selection just before the last-reward collection (at the 8th arm selection). In five successive trial sessions in unlesioned rats at the 8th arm selection, 0/30 cases occurred (same rats used in *Lesion experiments in well-trained rats*, before the lesion operation). After the lesion surgery, in AcbC-lesioned rats, it was also 0/30 cases. At the 9th arm selection, 33.3% (10/30) AcbC-lesioned rats showed a random pattern of arm selection. Compared to their performance before lesion surgery, they showed no significant difference (30%, 9/30 cases; P>0.05). Moreover, there was a significant drop in tip-approaching speed at the 9th approach compared to the 8th approach (Figure S5A; P<0.05, Sidak's multiple comparison test). We did observe, however, no significant decrease in platform-approaching speed just after the last-reward collection in the same AcbC-lesioned rats (Figure 3E; P>0.05, Sidak's multiple comparisons test).

Thus, lesioning the AcbC had a smaller effect on the rats' ability to predict the reward absence in each trial compared to their ability to control activity levels after the last-reward collection.

### Lesion of the AcbC did not change locomotor activity in the open-field test

**Figure 4:**
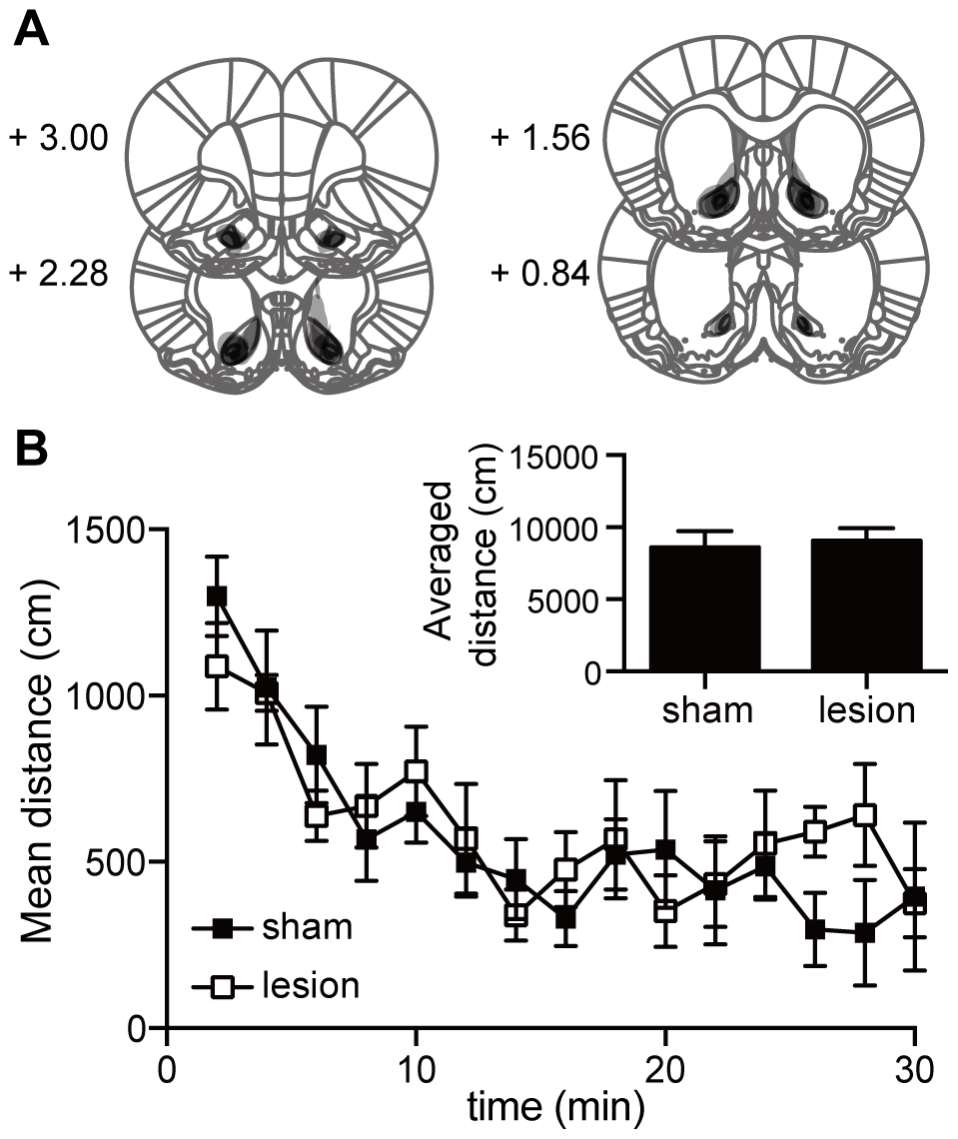
Effect of AcbC lesions on activity in open-field test. **A**: Extent of AcbC lesions in rats used in the open-field test. Dark areas indicate overlap of lesions among rats tested in the open field. Based on the histological analysis of rats in the 8-arm FFT, we selected 5 of 8 rats with comparable lesions in order to make valid comparisons of behavioral data with rats in other experiments. **B**: Change in traveling distance in the open arena, plotted at 2 min intervals. Inset graph shows total distance travelled during 30-minute test period. AcbC-lesioned rats (n = 5) showed no significant differences in activity level (t = 0.35, *P*>0.05, unpaired *t*-test) compared to sham controls (n = 5). Error bars denote SEM.

Was the increase in locomotor activity after AcbC lesions a non-specific elevation of general motor activity? To answer this question, we examined the influence of AcbC lesions on open-field activity. Five AcbC-lesioned rats and 5 sham-lesioned rats were allowed to run and explore a novel open field (Figure 4B). No food rewards were present.

AcbC-lesioned rats showed virtually the same level of activity as the control rats (Figure 4B, inset graph, P>0.05, unpaired t-test). The extent of damage in the AcbC (Figure 4A) overlapped considerably with the AcbC damage in rats receiving the lesion after training in the 8-arm FFT (Figure S6). Thus, enhanced motor activity of AcbC-lesioned rats in the 8-arm FFT is *not* likely a non-specific hyperactivity. This result argues that the AcbC mediates inhibition of actions motivated by rewards to regulate the activity level of planned resting behavior.

### Lesion of the AcbC before training impairs facilitation of platform-approaching speed

**Figure 5:**
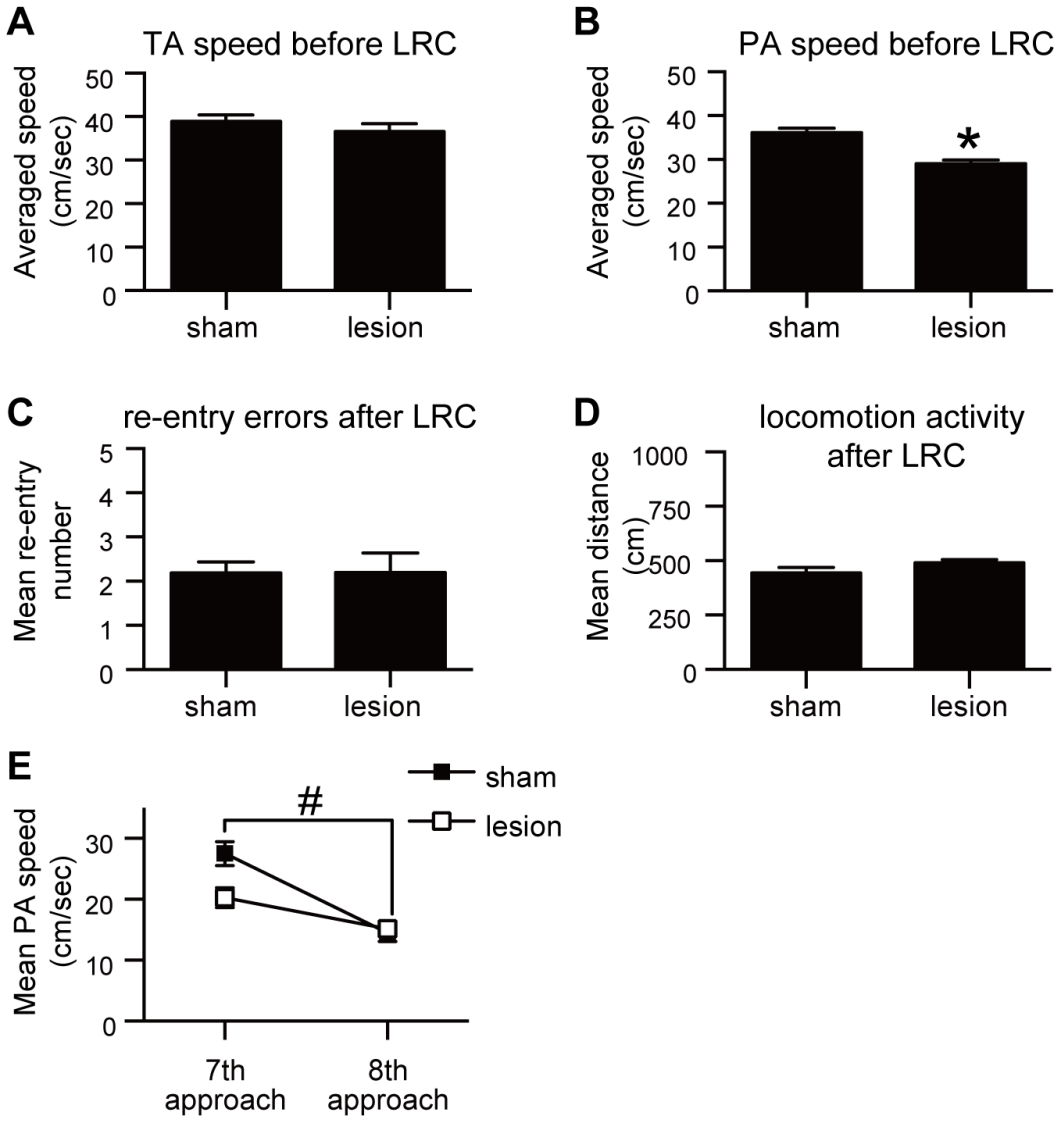
Effect of AcbC lesions on acquisition of optimized behavior in the 8-arm FFT. Experimentally naïve rats received lesions of the AcbC first, followed by training (AcbC-lesioned, n = 6; sham control, n = 6). Behavioral data from the 31st-35th trials were analyzed. **A**: Average tip-approaching (TA) speed before retrieving the last reward (LRC) in AcbC-lesioned rats did not differ compared to sham controls (t = 1.02, *P*>0.05, unpaired *t*-test). **B**: AcbC-lesioned rats showed decreased average platform-approaching (PA) speed before LRC compared to sham controls (t = 5.44, *P*<0.05, unpaired *t*-test). **C**: Number of re-entry errors after LRC in AcbC-lesioned rats and sham controls was not significantly different (t = 0.034, *P*>0.05, unpaired *t*-test). **D**: Locomotor activity in AcbC-lesioned rats after LRC was comparable to sham controls (t = 1.50, *P*>0.05, unpaired *t*-test). **E**: Mean Platform-approaching (PA) speed just before (7th arm visit) and just after (8th arm visit) LRC in AcbC-lesioned and sham-lesioned rats. AcbC-lesioned rats did not significantly decrease their platform-approaching speed just after LRC (*F*_1,5_ = 25.89, *P*<0.05, two-way ANOVA; AcbC-lesions, *P*>0.05, sham-lesions, *P*<0.05, Sidak's multiple comparison test). *P<0.05 compared to sham controls, #*P*<0.05 compared to 7th arm visit. Error bars denote SEM. LRC, the last-reward collection; GO, gate-open; TA, tip-approaching; PA, platform-approaching.

In the next experiment, we also investigated how AcbC lesions affect optimization of reward seeking behavior by using 8-arm FFT (Figure 5). Rats in this experiment received AcbC lesions before training on the 8-arm FFT (n = 6). Similar to the sham controls, AcbC-lesioned rats employed a sequential pattern of arm selection and had few re-entry errors before the last-reward collection (Figure S4B). Compared to sham-lesioned rats, AcbC-lesioned rats showed significant slowing in platform-approaching speeds during the period when rewards are available (Figure 5B; P<0.05, unpaired t-test). Their slow platform-approaching speed occurred right from the 1st arm traverse to the 7th arm traverse (Figure S7B; P<0.05). No significant difference in tip-approaching speeds was evident between the two groups in this period (Figure 5A, P>0.05). Analysis of these parameters from early stages to later stages of repetitive training (Figure S8) showed that the lesion effect on the platform-approaching speeds was evident across the 30 training trials. This suggests that the mechanism controlling tip- and platform-approaching speed is basically independent from each other, and that the AcbC contributes to facilitation of learning related to optimization of the reward-seeking behavior (platform-approaching speed).

### Lesion of the AcbC before training impairs facilitation of motivation to locomote after the last-reward collection

The histological analysis indicated that the animals used in this analysis received similar damage to the AcbC as rats lesioned after training (Figure S6; P>0.05). Rats that received lesions of the AcbC before training showed a similar number of arm re-entry errors (Figure 5C, P>0.05), and displayed a similar level of activity as the sham controls (Figure 5D, P>0.05) after the last-reward collection. On the other hand, in comparison to the rats lesioned after training (Figure 3), statistically significant reductions were observed in platform-approaching speed before the last-reward collection (t = 4.636, P<0.05, unpaired t-test), activity level (t = 6.581, P<0.05, unpaired t-test), and occurrence of re-entry errors after the last-reward collection (t = 3.594, P<0.05, unpaired t-test). These results show that the activity level in AcbC-lesioned rats before training shifts to a reduced level throughout the experimental period compared to rats lesioned after training.

Furthermore, similar to the well-trained rats that received AcbC lesions after training, rats receiving AcbC lesions before training did not show as much slowing at the 8th platform approach (Figure 5E, P>0.05, Sidak's multiple comparison test), although the sham control animals did (Figure 5E; P<0.05). It appears that lesion of the AcbC before training impairs the ability of the rat to reduce its activity level after the last-reward collection, similar to rats that received AcbC lesions after training when the global shift of activity level occurs, as mentioned above.

### Lesion of the AcbC affects the behavioral influence of the activity controller that determines activity levels in reward seeking and planned resting behavior

To examine whether the motivational mechanism controlling platform approaches before the last-reward collection affects the mechanism controlling locomotion after the last-reward collection, we analyzed the correlation between those two activities in intact rats who received over 30 trials of training (see subsection, *Experiments using normal adult rats* under *Description of methods and analyses for the different experimental groups* in Methods S1).

**Figure 6:**
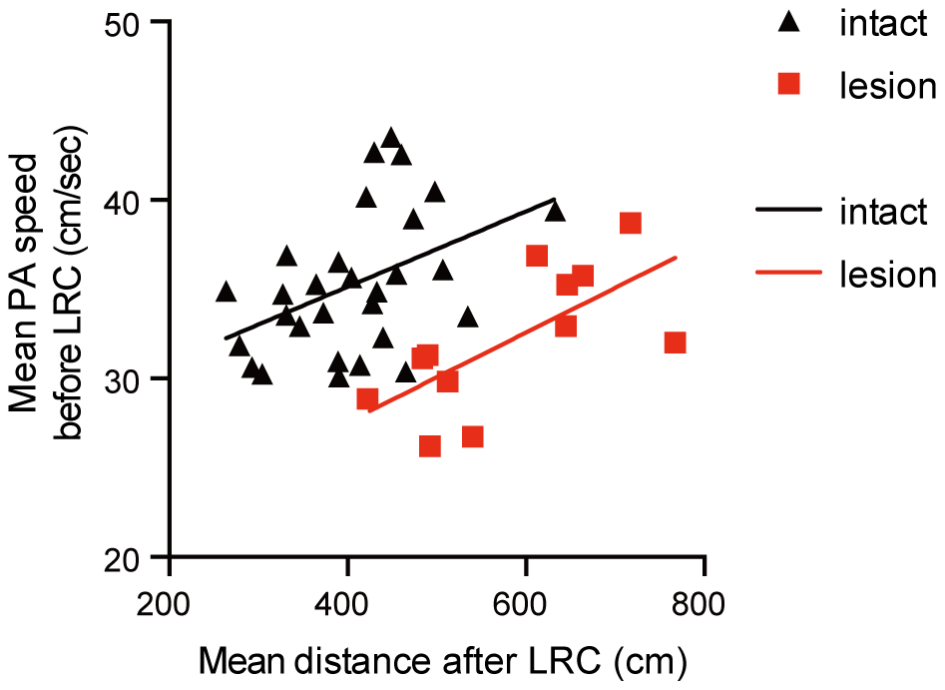
Activity level after the LRC correlates with platform-approaching speed preceding the LRC. Platform-approaching speeds before the LRC (reward-available period) and distances traveled after the LRC (reward-unavailable period) were analyzed using data obtained during the 31st-45th trials on the 8-arm FFT. Performance of AcbC-lesioned rats (n = 12) and intact, unlesioned rats (n = 30) were compared. Both groups showed statistically significant correlations between platform-approaching speeds before the LRC and activity level after the LRC (distances traveled) (intact group: r = +0.44, *P*<0.05; AcbC-lesioned group: r = +0.68, *P*<0.05), and the two regression lines were parallel (slopes; *P*>0.05). However, the regression line for the AcbC-lesion group shifted toward the right compared to the one for the intact group. Filled square and triangle symbols are average values for each rat. LRC, last-reward collection; PA, platform-approaching.

In intact animals (Figure 6), the distance traveled after the last-reward collection was significantly correlated with the platform-approaching speed observed before collecting the last reward (r = 0.44, P<0.05, slope = 0.021). This positive correlation suggests that the control mechanism regulating the rat's activity after eating the last reward shares a process with the one regulating platform-approaching speed before the last reward retrieved. That is, there is an activity controller that acts independently of the animal's prediction of a reward absence. This result also suggests that AcbC lesions mainly affect the activity controller's influence on behavior.

The scatter plots of distance traveled after the last-reward collection and platform-approaching speed observed before collecting the last reward are shown in Figure 6 for AcbC-lesioned rats. These also showed a significant positive correlation (r = 0.68, P<0.05), with a slope of the regression line similar to the one obtained for intact animals (slope = 0.025; P>0.05, compared to the slope of intact rat group). Furthermore, the regression line in AcbC-lesioned rats shifted to the right, showing a disinhibition effect caused by the lesion. That is, the AcbC specifically inhibits locomotion activity after reward absence. Thus, this positive and parallel correlation in AcbC-lesioned rats raises the possibility that activity controller function is maintained after damage to the AcbC.

## Discussion

Use of our 8-arm FFT in the present lesion study revealed the following interesting facts. Well-trained rats showed that they predicted a reward absence just after their last-reward collection and then started the planned resting behavior during the reward-unavailable period. Based on the results of making an AcbC lesion *after* training, we conclude that the AcbC inhibits the level of activity during the resting behavior. We also conclude that the AcbC is involved in motivational learning that sets the activity level of reward-seeking behavior (platform-approaching speed). This was observed in rats *trained after they had received AcbC lesions*.

We also examined the influence of AcbC lesions on exploration and habituation in an open-field test (Figure 4). However, there were no effects of AcbC lesions: Lesioned and unlesioned rats had similar patterns of activity in the open-field task, in which there was no reward learning. In the 8-arm FFT, we showed in intact rats that their motor activity after the last-reward collection is highly correlated with their platform-approaching speed during reward seeking (Figure 6), a behavior pattern that we suggest is inhibited by the AcbC. We also demonstrated that normal, unlesioned rats' running speeds in reward seeking behavior on the 8-arm FFT increase by repetitive training. Interestingly, there is a significant difference in the activity level after the last-reward collection in the two groups of AcbC-lesioned rats that show different degrees of motivational learning (i.e., rats that received AcbC lesions before or after training on the 8-arm-FFT). These results strongly suggest that target behavior for AcbC-mediated inhibition is controlled by a motivational mechanism affected by learning in the 8-arm FFT.

**Figure 7:**
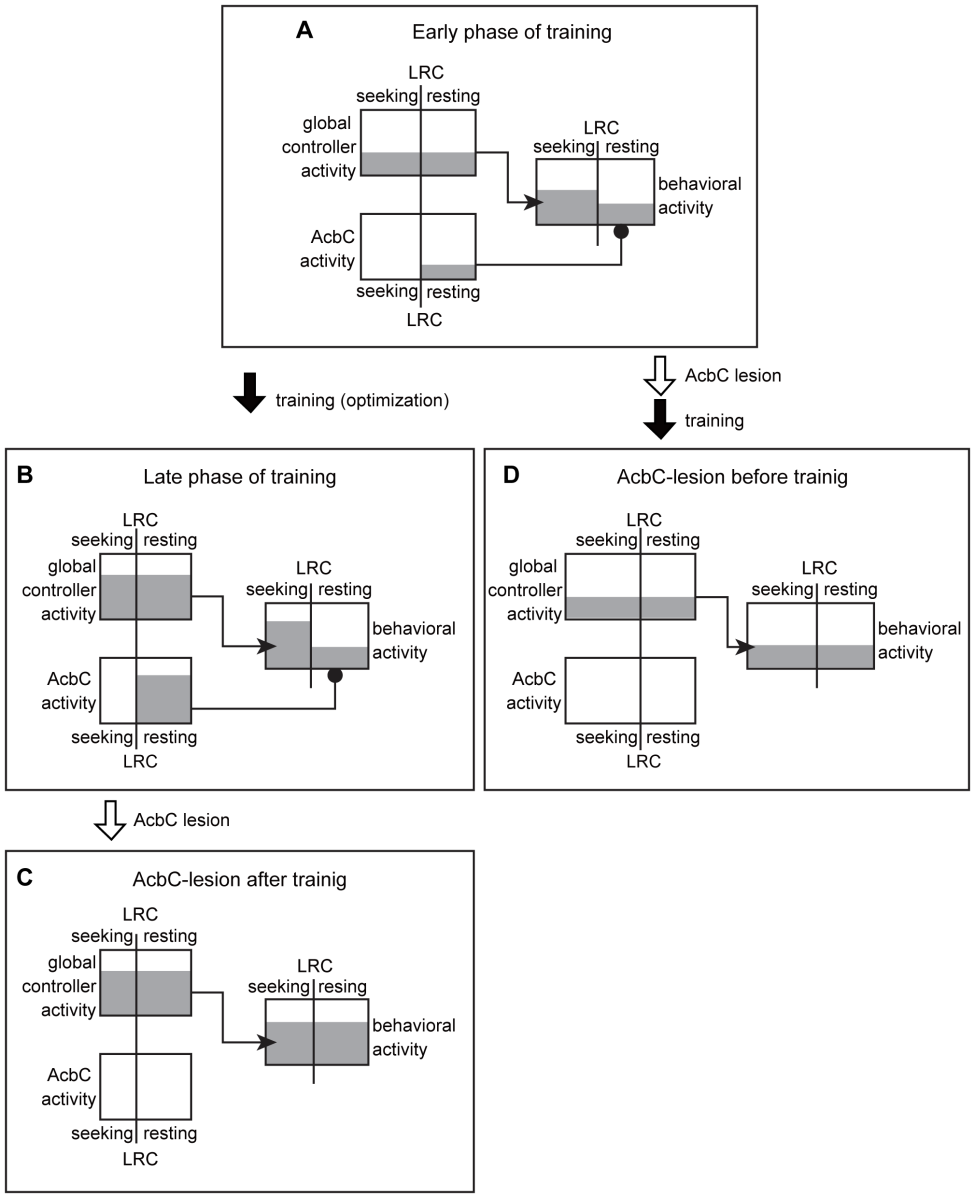
Schematic representation of hypothesized behavioral control according to changes in reward condition. From the presented results, we propose that the activity level in planned resting behavior is determined by intensity level of the global activity controller and the AcbC. The former regulates the level of platform-approaching behavior and behavior during the reward-unavailable period after the last-reward collection (LRC). The latter selectively controls the resting level in the reward-unavailable period after LRC. Height of gray highlighted areas represents intensity. **A**: In the early phase of 8-arm FFT training, the output intensities of the global activity controller and AcbC are relatively low. Thus, behavioral activity level (speed) of a platform approach before the LRC is relatively low, and the level of planned resting after LRC is not enough to inhibit redundant actions during the reward-unavailable period. **B**: After the repetitive training on the 8-arm FFT, the output intensities of global controller and AcbC are higher than those in early phase of training, contributing to the formation of a temporal behavior pattern that reflects the change in the reward situation. **C**: Lesioning of the AcbC after training enhances the activity level of planned resting after the LRC, because AcbC damage causes disinhibition. **D**: By contrast, repetitive training after lesion of AcbC fails to adjust the output level of the global controller to a higher state. This is because AcbC-mediated inhibition of (redundant) actions during the reward-unavailable period is required for learning to enhance the activity level of global controller.

Based on the results of the present study, we advance a provisional model for the mechanism of activity control mediated by the AcbC (Figure 7). First, we propose that the activity controller regulating platform-approaching speed before the last-reward collection also controls the level of basic activity after the last-reward collection. That is, the activity controller can be thought of as a global activity controller that is engaged during the entire task. Through repetitive training in the 8-arm FFT, the output of this global controller becomes greater than that occurring in early phases of training (Figure 7A and B). Next, we make the suggestion that the AcbC inhibits the output from the global activity controller after the last-reward collection in order to regulate the activity level of the planned resting behavior (Figure 7B). Therefore, lesioning of the AcbC after training causes disinhibition (Figure 7C). This reduction of activity by the AcbC contributes to inhibiting redundant actions that mismatch the behavioral context and as a consequence reduces behavioral cost during the reward-unavailable period. Third, we speculate that the temporally local reduction of behavioral cost through the action of the AcbC potentially affects the optimizing process of behavioral influence of the global activity controller. Therefore, lesioning of the AcbC before training fails to adjust the output level of the global controller to a higher state (Figure 7D).

It was already reported that some Acb neurons show greater firing levels when animals are in a “no task” state compared to periods when they are performing a task [30], [31]. Such neural activity in such a planned resting state is a candidate for the neural substrate that supplies inhibition from the AcbC. Based on these and our present results, we speculate that impairments of resting neural activity in human brains results from an increase of redundant behaviors that mismatch with a situation or context. This prevents optimization of behavior or decision-making. In frontotemporal dementia patients, for example, in which the salient network [32]–[34] that includes the Acb has degenerated, there is a tendency not to make decisions based on contexts relating to moral and sociality [35], [36]. Such decisions mismatched with context may, as a result, increase the frequency of stress for the patient. Elevated stress is one of the risk factors for dementia [37] and may contribute to progression of the disease.

Jongen-Relo et al. reported normal performance of AcbC-lesioned rats in a traditional place preference task [38]. In our task, there is a possibility that AcbC lesions affected the identification of where the final reward acquisition occurs, resulting in elevated locomotor activity in AcbC-lesioned rats after they retrieve the last reward. However, our results show that lesion of the AcbC *after* training has no affect on any aspects of reward-approaching behavior. Even with bilateral AcbC lesions before and after training, arm tip-approaching speed dropped when animals revisited the same, already rewarded arm (usually their 9th arm visit) (see Figure S5). We also showed that AcbC-lesioned rats could predict the location at which the last-reward collection occurred. In radial maze paradigms, impairment of Acb-lesioned rats in a working-memory paradigm (4-baits and 4-unbaits test) has been reported [38], [39]. It has also been reported that this impairment is caused by lesion of the Acb shell or inactivation of AMPA/kainate receptors but not Acb core damage [38], [39]. These previous studies support our conclusion that AcbC lesions do not affect rats' ability to use place recognition and working memory in an apparently normal way.

Previous work indicates that lesion of the Acb or AcbC results in hyperactivity in an open-field task [40]–[43]. It is, however, reported that lesion of the AcbC had no affect on locomotor activity in a *novel* open field [44]. We also showed here that lesion of the AcbC did not affect locomotor activity in the open-field task. We have no simple explanation for the discrepant results. Some possibilities are differences in the rearing of animals; in experimental conditions, such as food-restriction method; lighting conditions in experimental environment; and familiarization with the environment. We measured activity levels in the open-field test under bright-light conditions and over a relatively short-term assessment period (30 min), because our rats received 8-arm FFT training under bright-light conditions for several minutes per day. On the other hand, in our conditions, the rats that received training after AcbC lesioning were not hyperactive but rather hypoactive. Moreover, the rats receiving AcbC lesions after training showed the same activity level as controls in the waiting period before the arm gates opened in the 8-arm FFT. This occurred even when they showed hyperactivity after retrieving the last reward (data not shown). Thus, we believe that the effect of a general hyperactive tendency on the results is minimal.

The AcbC is strongly involved in activity or learning control of preparatory behaviors that have indirect commitment to reward acquisition [45]–[47]. The present results also show that the AcbC is critical for adaptive increase in the speed of platform approaches, an indirect kind of reward approach action. We also showed that AcbC is critical for determining the activity level in planned resting behavior, which is observed after the last-reward collection. Our analysis strongly suggested that the actions sensitive to lesion of the AcbC observed after the last-reward collection were also motivated by the prior rewards. As described above, we consider that one of the motivation systems driving behavior in 8-arm FFT is not sensitive to change of reward situations, and the AcbC temporally modulates its output to form situation dependency of behavior. Therefore, the AcbC was able to act as a gate for objective-oriented behaviors, as pointed out by Taha and Fields [30]. It also could enhance the optimizing process of the gated behaviors, as described here.

Many studies have reported that lesioning the AcbC *before* learning impairs instrumental responses [18] and Pavlovian behavior [48]. On the other hand, lesioning the AcbC after learning does not affect rats' behavior in the stop-signal task [49] and a forced-choice task [20]. These previous reports suggest that the Acb (especially, the AcbC) is important for the acquisition of learning, but not for the execution of learned behavior [50]. The present study using the 8-arm FFT demonstrates that the AcbC plays an important role in learning and executing a behavior. The differences observed in animals lesioned before and after learning may be partially masked by a difficulty in detecting how the lesion made after learning influences behavior. The effect of lesioning the AcbC on well-trained animals was detectable only when they did not perform purposeful behaviors that were usually focused in traditional behavioral experiments.

It has been reported that DA depletion decreases locomotor activity [51], [52]. It is also reported that by lesioning or inactivating the AcbC, rats tend to select impulsive [17], [53] or risk-aversive behaviors. Some studies reported that, when animals are given some choices, including a risky one, activity of DA neurons in the ventral tegmental area and DA release in the AcbC encodes information about better (optimal) options, even if animals actually chose an option [54]–[56]. On the other hand, Kelsey and Willmore [57] pointed out that the role of the Acb output is to inhibit locomotion, whereas the role of DA input to the Acb is to enhance locomotion by disinhibiting Acb output.

In conclusion, we propose that at least part of the AcbC output activity is utilized to inhibit actions in a situation-dependent manner. As shown here, these actions were redundant for a specific situation. This would contribute to the formation of a temporal gate for a purposeful behavior set.

## Supporting Information

Figure S1 **Examples of sequential and non-sequential patterns of arm selection.** Solid, irregular lines superimposed on schematic of 8-arm maze show movement traces of an example rat's traverses up and down the arms. Lowercase letters at arm tips indicate the order of arm selections. Repetitive training on the 8-arm FFT resulted in rats adopting a sequential pattern of arm selections (A). In this pattern, each successive turn of the rat in the trial is 45°. With a random arm-selection pattern (B), the rat selects a non-adjacent arm at some point(s) in the trial. In this instance, the 3rd visited arm (c) was *not* adjacent to the 4th visited arm (d). a, 1st selected arm; b, 2nd selected arm; c, 3rd selected arm; d, 4th selected arm; e, 5th selected arm; f, 6th selected arm; g, 7th selected arm; h, 8th selected arm.
(TIF)

Figure S2 **Change in the occurrence of random arm-selection patterns before the last-reward collection.** To clarify the learning process, we trained 10 adult rats for 50 trials on the 8-arm FFT. Repetitive training on the 8-arm FFT decreased the occurrence of random arm-selection patterns before the last-reward collection (see subsection about sequential patterns of arm selection, Analysis of arm-selection patterns in MATERIALS & METHODS). Data represent means ±SEM.
(TIF)

Figure S3 **Behavioral performance of well-trained rats assessed in five successive trials before AcbC lesions.** Before AcbC- (n = 6) or sham-lesion surgery (n = 6), both groups attained similar performance levels in the 8-arm FFT. Tip-approaching (TA) (**A**) and platform-approaching (PA) speeds (**B**), the number of re-entry errors (**C**), and the traveling distance (**D**) after the last-reward collection (LRC) of the two groups were not statistically different (**A**, *t* = 1.18; **B**, *t* = 0.58; **C**, *t* = 0.98; **D**, *t* = 0.93; all *P*>0.05, unpaired t-test). Data represent means ±SEM.
(TIF)

Figure S4 **Effect of AcbC lesions on re-entry errors before the last-reward collection.** We examined the effects of AcbC lesions on well-trained rats (rats lesioned *after* training, n = 6; sham-lesioned rats, n = 6) and untrained rats (rats lesioned *before* training, n = 6; sham-lesioned rats, n = 6). Lesioning of the AcbC had no effects on re-entry errors before the last-reward collection. Data represent means ±SEM.
(TIF)

Figure S5 **Effect of AcbC lesions on tip-approaching speed just before and just after the last-reward collection.** We compared the effects of AcbC lesions on the tip-approaching (TA) speed measured between two points: just before (8th arm approach) and just after (9th arm approach) the last-reward collection (LRC). AcbC-lesioned rats that received lesions after training (lesions, n = 6; sham control rats, n = 6) (A) and others that received lesions before training (lesions, n = 6; sham control rats, n = 6) (B) showed significant decreases in tip-approaching speed at the 9th arm approach (lesioned after training, *F*_1,11_ = 2.76; lesioned before training, *F*_1,11_ = 192.7; all *P*<0.05, Sidak's multiple comparison test). #*P*<0.05 compared to TA speed on the 8th arm visit. Error bars denote SEM.
(TIF)

Figure S6 **Histological analysis of lesioned groups.** Comparison of the percentage of AcbC that was damaged, as assessed in coronal sections, in rats lesioned after training in the 8-arm FFT (dotted line), rats lesioned before training (solid line), and rats lesioned before the open-field test (broken line). There were no significant differences in the extent or distribution of the lesioned areas across the three groups (*F*_2,16_ = 2.91, *P*>0.05, two-way ANOVA). Error bars denote SEM.
(TIF)

Figure S7 **Detailed analysis of running speed of reward-seeking in rats that received AcbC lesion before training.** We compared the effects of lesioning the AcbC *before* training on the tip-approaching (TA) speed and platform-approaching (PA) speed before the last-reward collection (LRC). AcbC-lesioned rats showed no significant differences in tip-approaching speed (**A**) compared to sham control rats (*F*_1,10_ = 2.23, *P*>0.05, two-way ANOVA). However, rats receiving AcbC lesions *before* training showed slower platform-approaching speeds (**B**) on the 1st arm traverse to the 7th arm traverse than sham controls (*F*_1,10_ = 29.17, *P*<0.05, two-way ANOVA). Error bars denote SEM.
(TIF)

Figure S8 **Quantitation of running speed changes in AcbC-lesioned rats during learning of tip-approaching and platform-approaching before the last-reward collection.** As with normal rats, the behavioral performance parameters of rats that received lesions of the AcbC before repetitive training on the 8-arm FFT plateaued before the 30th trial. A: Change in tip-approaching (TA) speed before the last-reward collection (LRC). B: Change in platform-approaching (PA) speed before LRC. Error bars denote SEM.
(TIF)

Methods S1 **Supplementary methods.**
(DOC)
