# Supplementary figures and images for: Contribution of Nucleus Accumbens Core (AcbC) to Behavior Control during a Learned Resting Period: Introduction of a Novel Task and Lesion Experiments
Source: PLoS One. 2014 Apr 28;9(4):e95941. doi: 10.1371/journal.pone.0095941 (PMC4002452; doi:10.1371/journal.pone.0095941)

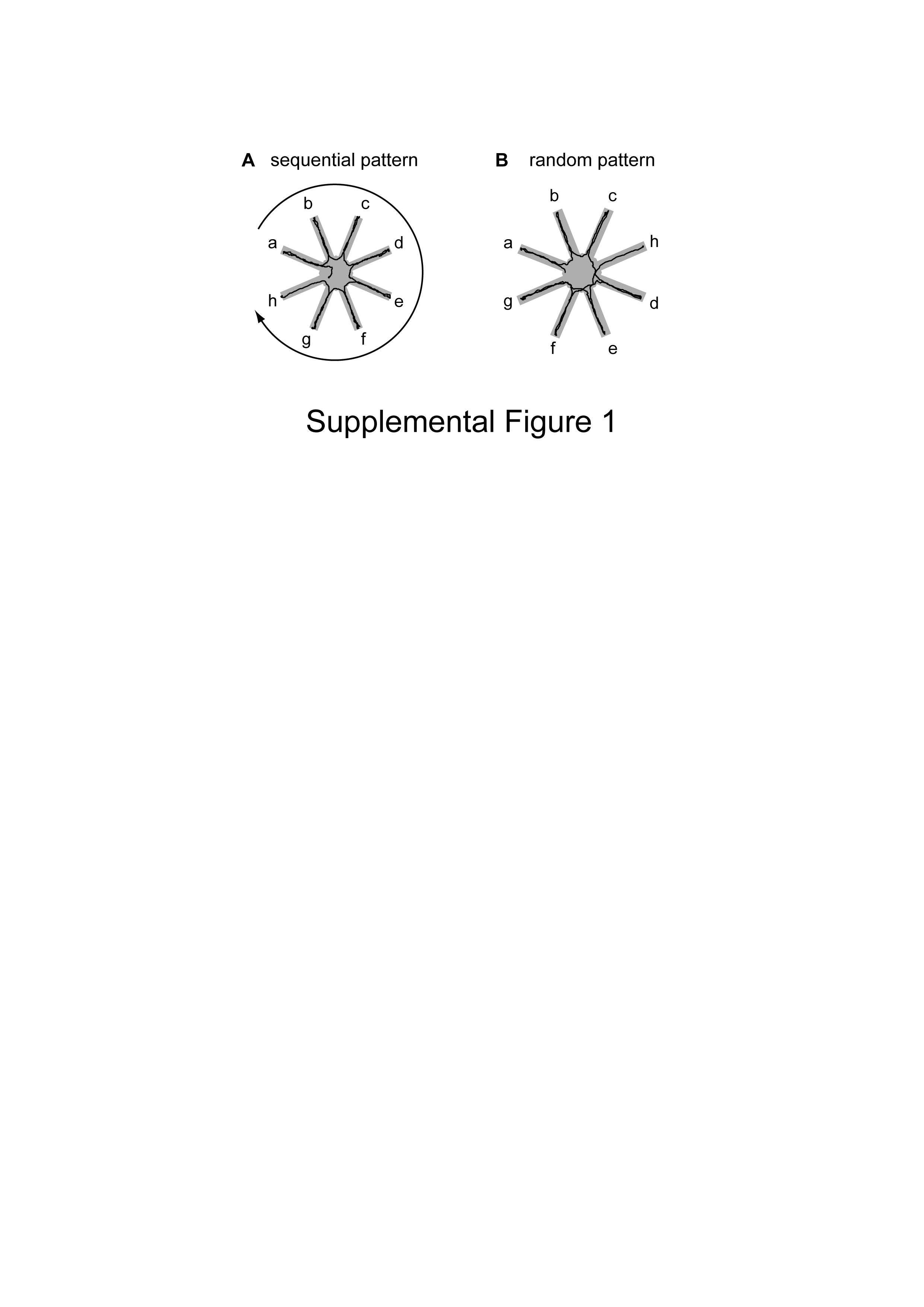

Supplement: Figure S1 — Examples of sequential and non-sequential patterns of arm selection. Solid, irregular lines superimposed on schematic of 8-arm maze show movement traces of an example rat's traverses up and down the arms. Lowercase letters at arm tips indicate the order of arm selections. Repetitive training on the 8-arm FFT resulted in rats adopting a sequential pattern of arm selections (A). In this pattern, each successive turn of the rat in the trial is 45°. With a random arm-selection pattern (B), the rat selects a non-adjacent arm at some point(s) in the trial. In this instance, the 3rd visited arm (c) was not adjacent to the 4th visited arm (d). a, 1st selected arm; b, 2nd selected arm; c, 3rd selected arm; d, 4th selected arm; e, 5th selected arm; f, 6th selected arm; g, 7th selected arm; h, 8th selected arm. (TIF) [file pone.0095941.s001.tif]

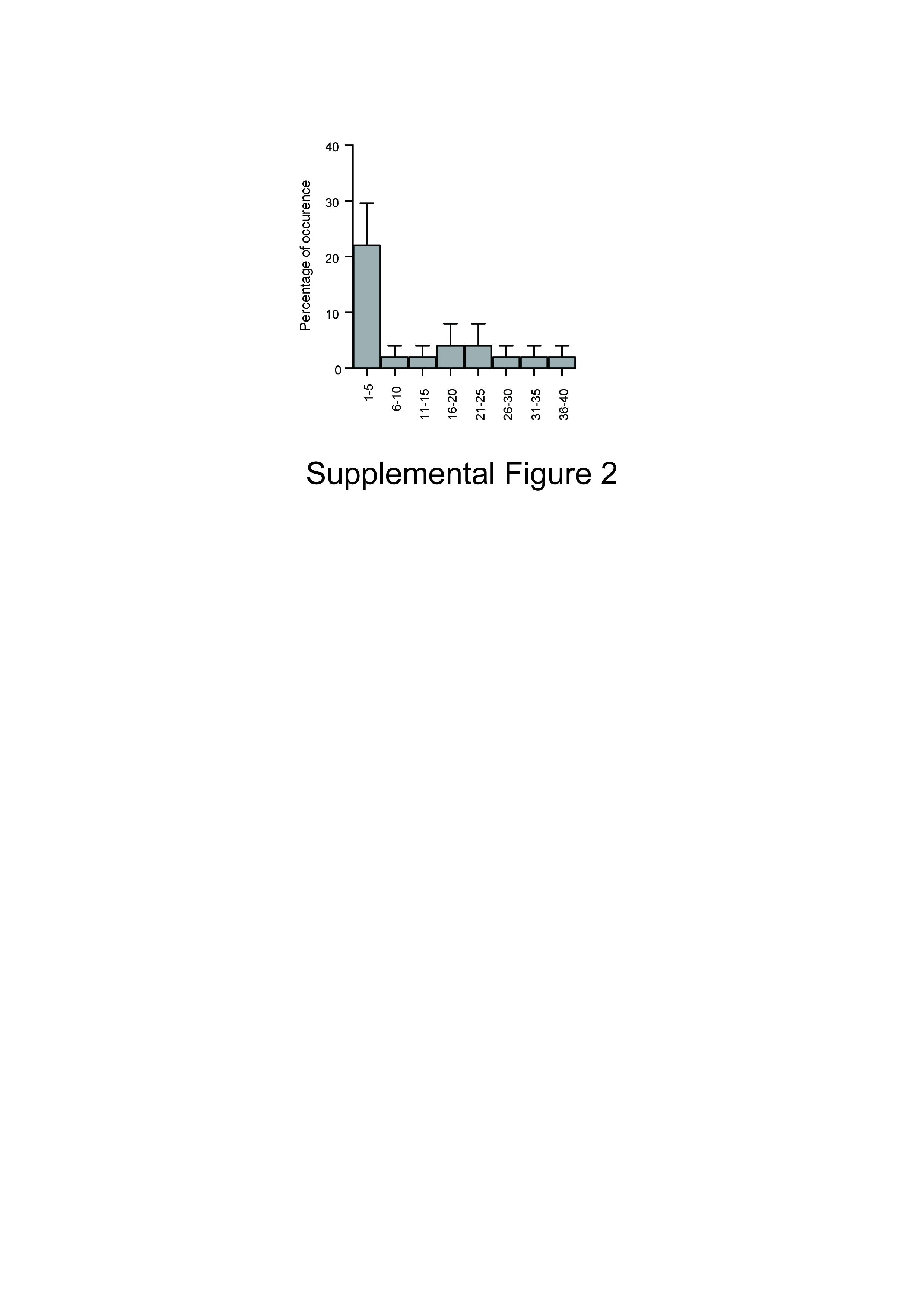

Supplement: Figure S2 — Change in the occurrence of random arm-selection patterns before the last-reward collection. To clarify the learning process, we trained 10 adult rats for 50 trials on the 8-arm FFT. Repetitive training on the 8-arm FFT decreased the occurrence of random arm-selection patterns before the last-reward collection (see subsection about sequential patterns of arm selection, Analysis of arm-selection patterns in MATERIALS & METHODS). Data represent means ±SEM. (TIF) [file pone.0095941.s002.tif]

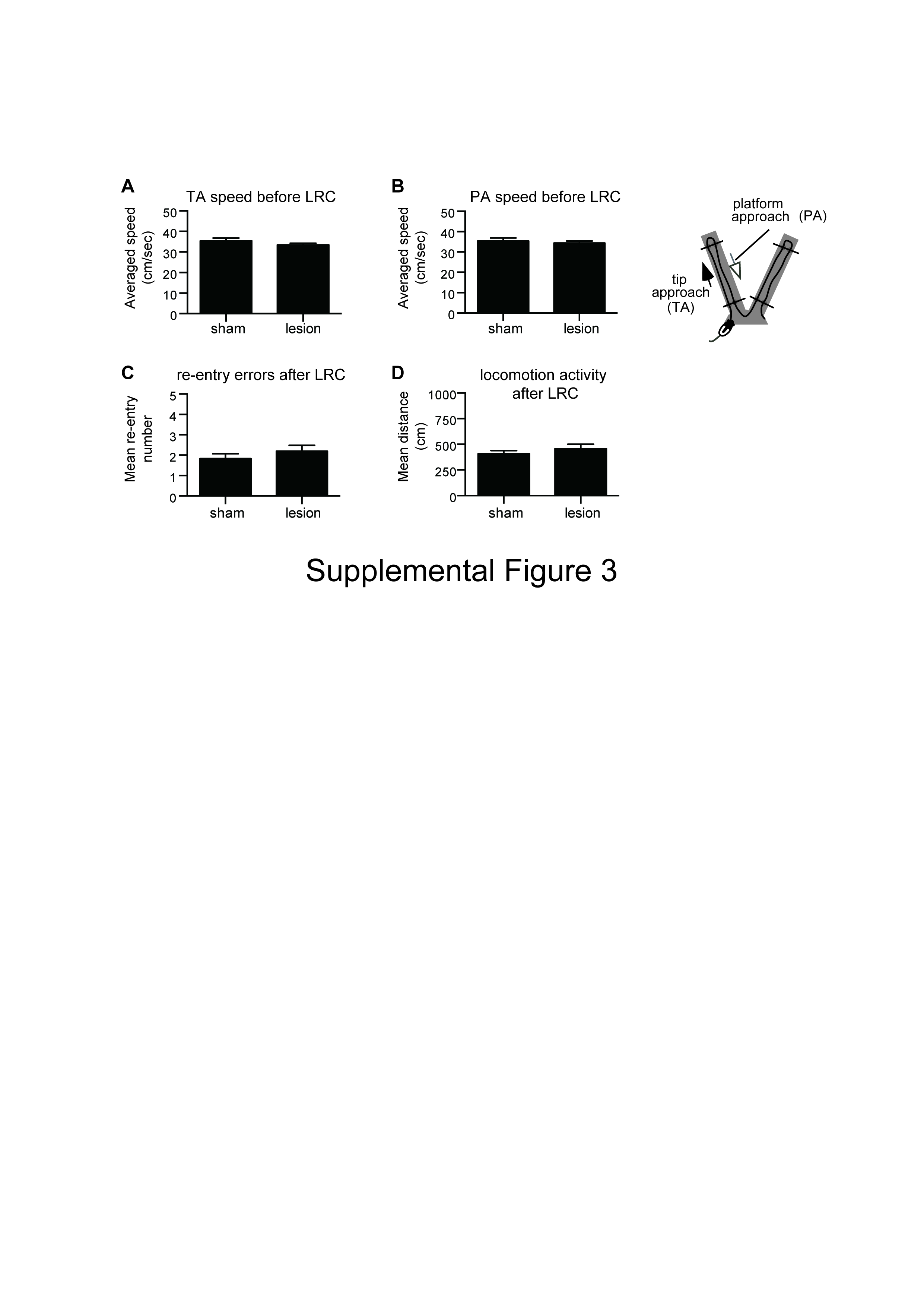

Supplement: Figure S3 — Behavioral performance of well-trained rats assessed in five successive trials before AcbC lesions. Before AcbC- (n = 6) or sham-lesion surgery (n = 6), both groups attained similar performance levels in the 8-arm FFT. Tip-approaching (TA) (A) and platform-approaching (PA) speeds (B), the number of re-entry errors (C), and the traveling distance (D) after the last-reward collection (LRC) of the two groups were not statistically different (A, t = 1.18; B, t = 0.58; C, t = 0.98; D, t = 0.93; all P>0.05, unpaired t-test). Data represent means ±SEM. (TIF) [file pone.0095941.s003.tif]

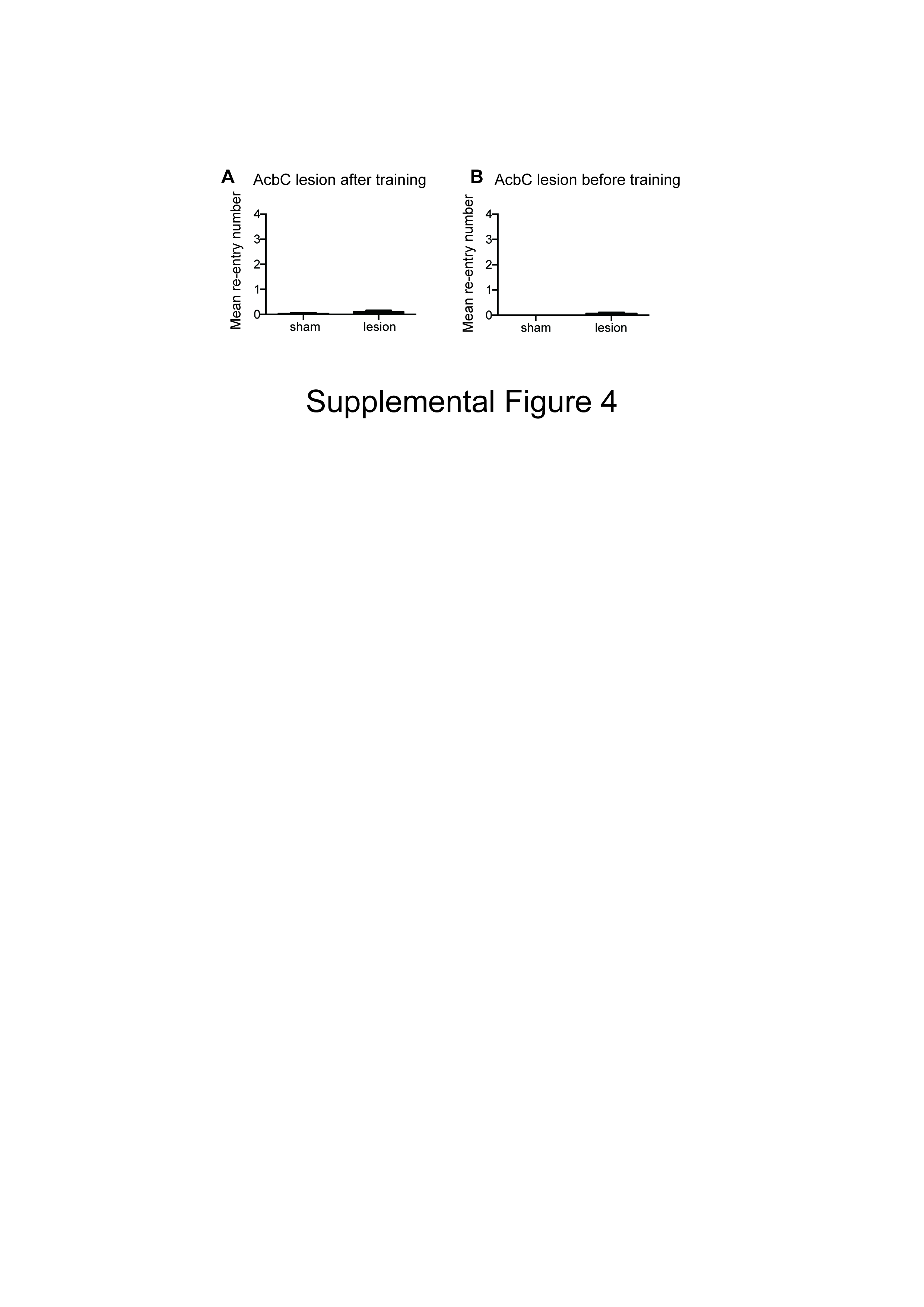

Supplement: Figure S4 — Effect of AcbC lesions on re-entry errors before the last-reward collection. We examined the effects of AcbC lesions on well-trained rats (rats lesioned after training, n = 6; sham-lesioned rats, n = 6) and untrained rats (rats lesioned before training, n = 6; sham-lesioned rats, n = 6). Lesioning of the AcbC had no effects on re-entry errors before the last-reward collection. Data represent means ±SEM. (TIF) [file pone.0095941.s004.tif]

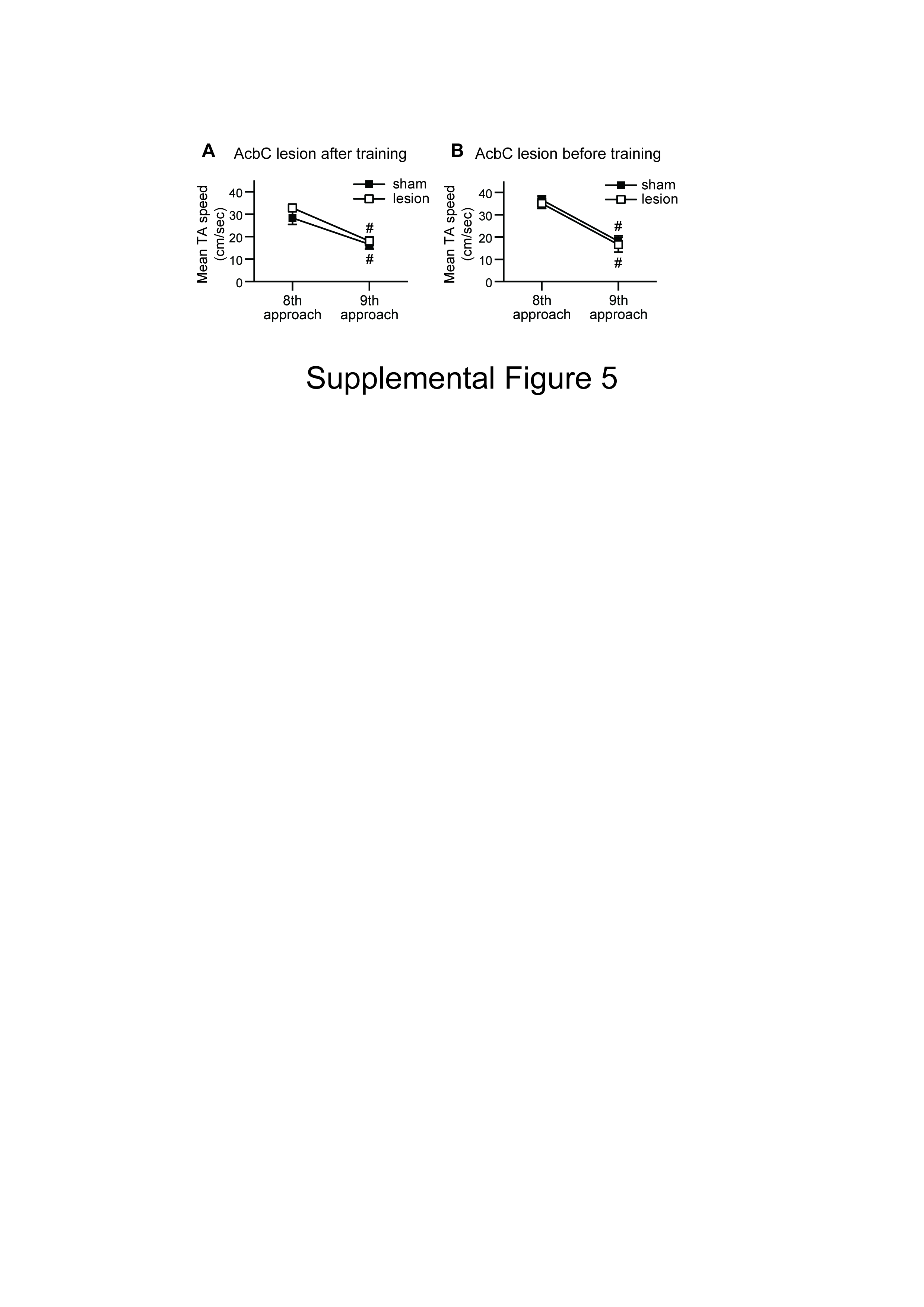

Supplement: Figure S5 — Effect of AcbC lesions on tip-approaching speed just before and just after the last-reward collection. We compared the effects of AcbC lesions on the tip-approaching (TA) speed measured between two points: just before (8th arm approach) and just after (9th arm approach) the last-reward collection (LRC). AcbC-lesioned rats that received lesions after training (lesions, n = 6; sham control rats, n = 6) (A) and others that received lesions before training (lesions, n = 6; sham control rats, n = 6) (B) showed significant decreases in tip-approaching speed at the 9th arm approach (lesioned after training, F 1,11 = 2.76; lesioned before training, F 1,11 = 192.7; all P<0.05, Sidak's multiple comparison test). #P<0.05 compared to TA speed on the 8th arm visit. Error bars denote SEM. (TIF) [file pone.0095941.s005.tif]

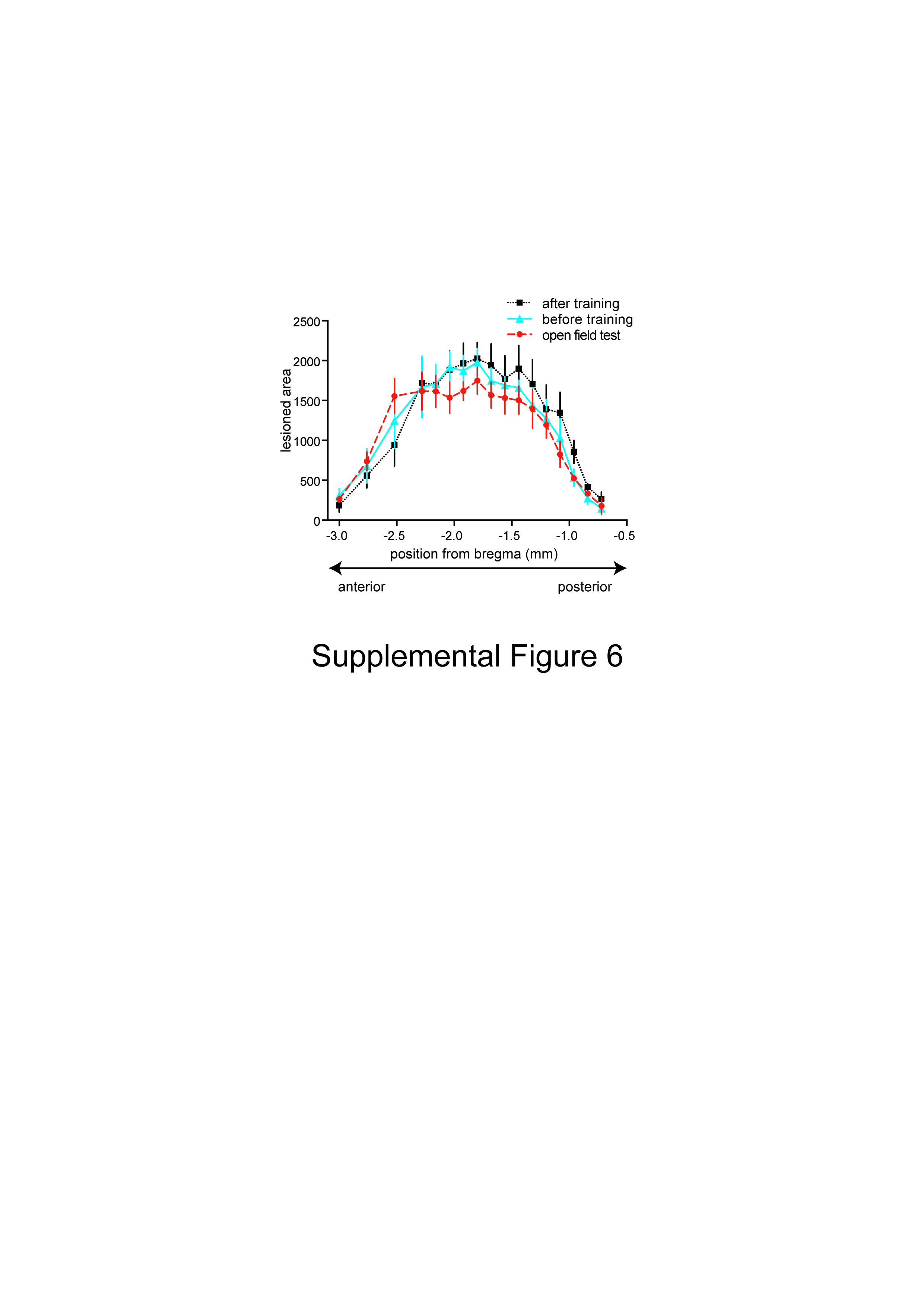

Supplement: Figure S6 — Histological analysis of lesioned groups. Comparison of the percentage of AcbC that was damaged, as assessed in coronal sections, in rats lesioned after training in the 8-arm FFT (dotted line), rats lesioned before training (solid line), and rats lesioned before the open-field test (broken line). There were no significant differences in the extent or distribution of the lesioned areas across the three groups (F 2,16 = 2.91, P>0.05, two-way ANOVA). Error bars denote SEM. (TIF) [file pone.0095941.s006.tif]

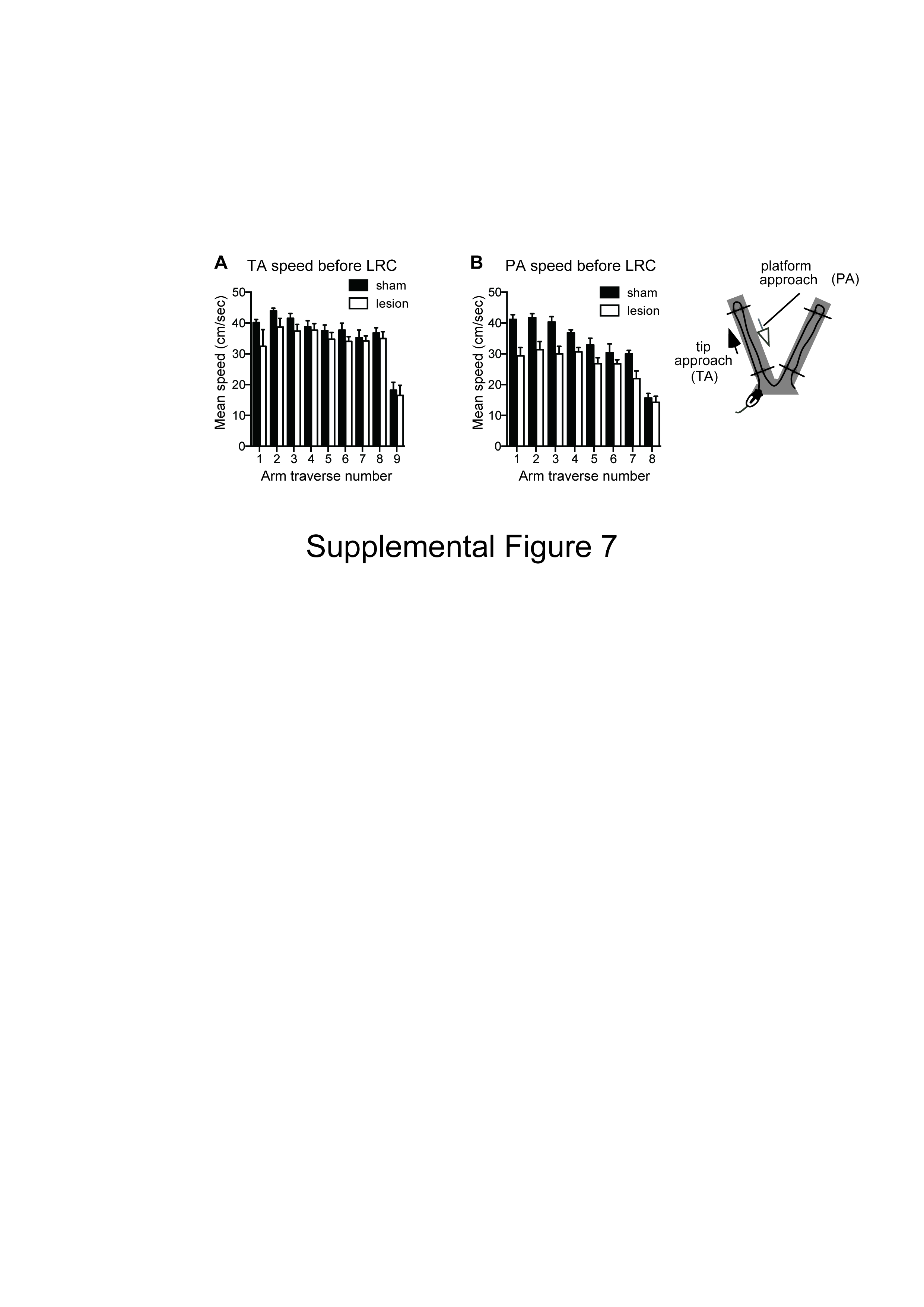

Supplement: Figure S7 — Detailed analysis of running speed of reward-seeking in rats that received AcbC lesion before training. We compared the effects of lesioning the AcbC before training on the tip-approaching (TA) speed and platform-approaching (PA) speed before the last-reward collection (LRC). AcbC-lesioned rats showed no significant differences in tip-approaching speed (A) compared to sham control rats (F 1,10 = 2.23, P>0.05, two-way ANOVA). However, rats receiving AcbC lesions before training showed slower platform-approaching speeds (B) on the 1st arm traverse to the 7th arm traverse than sham controls (F 1,10 = 29.17, P<0.05, two-way ANOVA). Error bars denote SEM. (TIF) [file pone.0095941.s007.tif]

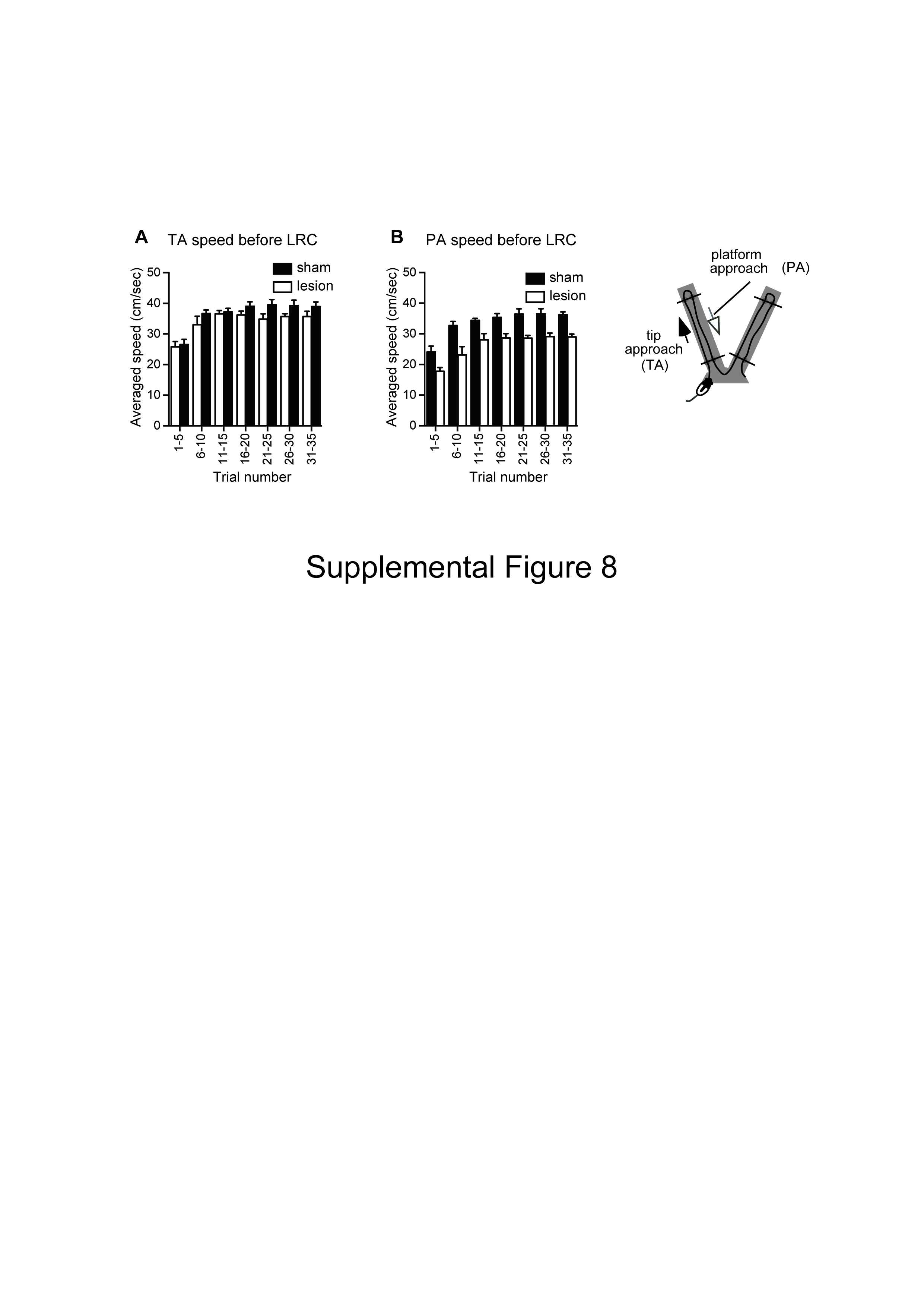

Supplement: Figure S8 — Quantitation of running speed changes in AcbC-lesioned rats during learning of tip-approaching and platform-approaching before the last-reward collection. As with normal rats, the behavioral performance parameters of rats that received lesions of the AcbC before repetitive training on the 8-arm FFT plateaued before the 30th trial. A: Change in tip-approaching (TA) speed before the last-reward collection (LRC). B: Change in platform-approaching (PA) speed before LRC. Error bars denote SEM. (TIF) [file pone.0095941.s008.tif]
