# Supplementary material for: Contribution of Nucleus Accumbens Core (AcbC) to Behavior Control during a Learned Resting Period: Introduction of a Novel Task and Lesion Experiments
Source: PLoS One. 2014 Apr 28;9(4):e95941. doi: 10.1371/journal.pone.0095941 (PMC4002452; doi:10.1371/journal.pone.0095941)
Supplement: Methods S1 — Supplementary methods. (DOC) [file pone.0095941.s009.doc]

**Supplementary Methods**

**Description of methods and analyses for the different experimental groups**

Basically, different groups of rats were used for the different experiments and analyses. Some data, or portions of data of different groups, were re-used to answer some questions that arose. Normal adult rats that never received lesions were used to examine learning on the new 8-arm FFT and optimization of behavioral actions. Other groups of adult rats received AcbC or sham lesions either before or after training on the 8-arm FFT. The methods and analyses for these different conditions are described in more detail in the subsections that follow.

*Experiments using normal adult rats*

Animals: To clarify the learning process by which experimentally naïve, adult rats shape their optimized behavior, 10 adult rats received 50 trials on the 8-arm FFT. These rats never received lesions. Besides these 10 rats, an additional 16 naïve adult rats received 36-45 trials on the 8-arm FFT. The latter 16 rats received lesions after the 40th training trial for other experiments.

Data collection: We calculated the rats’ traveling distance, arm-selection patterns, number of re-entry errors, and tip-approaching and platform-approaching speeds on each arm traverse for five successive trials using the 10 normal adult rats. Part of the data obtained from these 10 normal animals was used for determining how behavior in the 8-arm FFT is optimized in well-trained rats. These data are shown in Figure 2 (standard learning process).

Correlation analysis: For detailed analyses of normal rats at different learning stages on the 8-arm FFT (Figure 6), we re-used speed and activity level data from the different experimental groups. For this analysis, all data came from normal adult rats that had not yet received lesions for their experiment, or that would never receive lesions. Performance in two operationally defined learning stages was examined: (1) 31st-35th trials as a control for the rats receiving AcbC lesions before training, and (2) 41st-45th trials as a control for the rats receiving AcbC lesions after training. Data for the first stage came from two groups of rats: (1) ones that received about 40 trials of 8-arm FFT training (n=10; these eventually received AcbC lesions after the 40 trials used for this analysis); and (2) ones that received 50 trials on the 8-arm FFT only (n=10; these were the 10 normal adult rats described in the previous section, who were used to clarify the learning process). Thus, 20 normal adult rats contributed data to this analysis of the first group. Data from the 10 normal adult rats also contributed data to the second stage (41st-45th trials). To compare the intact rats and the AcbC-lesioned rats, a group that consisted of rats in different learning stages (6 received lesions *after* training, and 6 rats received lesions *before* training [n=12]), we combined these two groups (31-35th trials [n=20] + 41-45th trials [n=10]).

*Lesion experiments in well-trained rats*

Animals and lesions: These experiments used rats that first received training in the 8-arm FFT, followed by lesioning of the AcbC. Thus, we could examine the role of the AcbC in execution of already optimized behavior in this task. Naïve adult rats (n=10) first received 36-45 trials (mean = 40.3) on the 8-arm FFT, and then underwent surgery for bilateral lesions of the AcbC. On the basis of the histological analysis (Fig. 1), we selected 6 of the 10 rats for further analysis of behavioral performance (the lesioned areas of the 3 other rats were smaller than intended and included mainly the more rostral Acb; thus, they were excluded from further analysis).

For a sham lesion control group, six rats received bilateral microinjections of vehicle (PBS) after receiving 36-45 trials (mean = 42.0) on the 8-arm FFT.

Data collection: For rats with sham or AcbC lesions, we calculated the same behavioral parameters as we did with the normal group (see section, *Experiments using normal adult rats*). Data were also analyzed for 5 successive trials after the rats recovered from the lesions. The occurrence of the first random arm selection was performed as described above (see section, *Analysis of random arm selection*).

*Lesion experiments in naïve, untrained rats*

Animals and lesions: These experiments used rats that first received lesions of the AcbC, followed by training in the 8-arm FFT. Thus, these experiments examined how the AcbC contributes to establishing optimized reward-seeking actions in the 8-arm FFT. Ten naïve adult rats first underwent surgery for bilateral lesions of the AcbC. Six additional rats received sham control lesions. After surgical recovery, all lesioned rats received 35 trials on the 8-arm FFT.

On the basis of histological results showing the completeness of the AcbC lesions, 6 animals were selected from the AcbC-lesion group for further behavioral analysis (Fig. 1). We excluded from analysis the remaining 4 rats, which had smaller areas of the AcbC lesioned.

Data collection: The same behavioral parameters were calculated as before (see section, *Experiments using normal adult rats*). In this analysis, we analyzed data from the 31st-35th trials.

*Analysis using open-field task*

Animals and lesions: We used 13 experimentally naïve rats that received lesions *before* testing in the open-field apparatus. Thus, we could examine whether comparable damage to the AcbC causes general changes in locomotor/exploratory behavior. Eight rats received bilateral lesions of the AcbC, and 5 rats received sham lesions of the AcbC. On the basis of the histological analysis (Fig. 4A), for this analysis we selected 5 of 8 rats that received lesions of the AcbC comparable in size to the ones in lesioned rats trained in the 8-arm FFT in order to make valid comparisons (Supplementary Fig. S5). These rats are different from the ones tested on the 8-arm FFT.

Data collection: We calculated the lesioned rats’ traveling distance on the arena surface of the open-field task for one session (30 minutes). Total distance traveled was determined every 2 min for 30 min total. This produced a rough estimate of general locomotor activity.
